# Prenatal exposure to opioids and neurodevelopment in infancy and childhood: A systematic review

**DOI:** 10.3389/fped.2023.1071889

**Published:** 2023-02-21

**Authors:** Arin A. Balalian, Richard Graeve, Matthias Richter, Astrid Fink, Heike Kielstein, Silvia S. Martins, Morgan M. Philbin, Pam Factor-Litvak

**Affiliations:** ^1^ Department of Epidemiology, Mailman School of Public Health, Columbia University, New York, NY, United States; ^2^ Medical Faculty, Martin Luther University Halle-Wittenberg, Halle (Saale), Germany; ^3^ Social Determinants of Health Group, Department of Sport and Health Sciences, Technical University of Munich (TUM), Germany; ^4^ Department of Health and Consumer Protection, Kreis Groß-Gerau, Groß-Gerau, Germany; ^5^ Institut für Anatomie und Zellbiologie, Martin-Luther-Universität Halle, Halle (Saale), Germany; ^6^ Department of Sociomedical Sciences, Mailman School of Public Health, Columbia University, New York, NY, United States

**Keywords:** neurodevelopment, opioids, *in utero* exposure, prenatal exposure delayed effects, opioid-Related disorders

## Abstract

**Aim:**

This systematic review aims to estimate the relationship between prenatal exposure to opioids and neurodevelopmental outcomes and examines potential sources of heterogeneity between the studies.

**Methods:**

We searched four databases through May 21st, 2022: PubMed, Embase, PsycInfo and the Web of Science according to a specified search strings. Study inclusion criteria include: (1) cohort and case-control peer-reviewed studies published in English; (2) studies comparing neurodevelopmental outcomes among children with prenatal opioid-exposure (prescribed or used non-medically) vs. an unexposed group. Studies investigating fetal alcohol syndrome or a different primary prenatal exposure other than opioids were excluded. Two main performed data extraction using “Covidence” systematic review platform. This systematic review was conducted in accordance with PRISMA guidelines. The Newcastle-Ottawa-Scale was used for quality assessment of the studies. Studies were synthesized based on the type of neurodevelopmental outcome and the instrument used to assess neurodevelopment.

**Results:**

Data were extracted from 79 studies. We found significant heterogeneity between studies due to their use of different instruments to explore cognitive skills, motor, and behavioral outcomes among children of different ages. The other sources of heterogeneity included: procedures to assess prenatal exposure to opioids; period of pregnancy in which exposure was assessed; type of opioids assessed (non-medical, medication used for opioid use dis-order, prescribed by health professional), types of co-exposure; source of selection of prenatally exposed study participants and comparison groups; and methods to address lack of comparability between exposed and unexposed groups. Cognitive and motor skills as well as behavior were generally negatively affected by prenatal opioid exposure, but the significant heterogeneity precluded a meta-analysis.

**Conclusion:**

We explored sources of heterogeneity in the studies assessing the association between prenatal exposure to opioids and neurodevelopmental outcomes. Sources of heterogeneity included different approaches to participant recruitment as well as exposure and outcome ascertainment methods. Nonetheless, overall negative trends were observed between prenatal opioid exposure and neuro-developmental outcomes.

## 1. Introduction

An estimated 61 million people used opioids worldwide in 2020, a class of drugs that includes heroin, opium (opiates), and other synthetic opioids, including the pharmaceutical opioids used for non-medical purposes. This worldwide and common use poses a large threat to public health (1). Opioid use accounts for the vast majority of years of healthy lives lost due to substance use worldwide (1). In 2020, the prevalence of non-medical opioid use in North America was 3.37 percent among the 15–64 year old population, which is significantly higher than 2020 global prevalence of 1.2 percent and European prevalence of 0.59 percent among the same aged population (1).

Only one in five patients who receive medication for a drug use disorder is female, while one third of people who use drugs are women (1). In the US, the rate of opioid use disorder (OUD) among pregnant women at time of delivery increased fourfold from 1.5 cases to 6.5 cases per 1,000 delivery hospitalizations between 1999 and 2014, respectively (2). It is estimated that 14% to 22% of women receive an opioid prescription during pregnancy (3). The prenatal period represents an important period for neurodevelopment as the brain is morphologically developing, forming the basis for cognitive, motor and behavioral function (4). It is therefore important to study the impacts of in-utero opioid exposure on infant outcomes. Existing research in human brain cell cultures, suggests that opioids induce apoptosis (5) and in rodent models opioids are associated with impaired neurotransmitter uptake (6, 7). Similarly, the rodents exposed to opioids tend to have impaired learning and memory skills (8, 9). Multiple mechanisms could be associated with altered neurodevelopmental outcomes (10). In rodents, prenatal exposure to opioids were found to be associated with shorter dendritic lengths in somatosensory cortex (11) decreased neuronal proliferation (12) and increased apoptosis in dopaminergic cells.

Opioid use during pregnancy often results in Neonatal Opioid Withdrawal Syndrome (NOWS) in the newborn. NOWS is a cluster of symptoms which includes hyperactivity, difficulty feeding, irritability, vomiting, and diarrhea. Newborns are often treated for NOWS with medications such as morphine or buprenorphine (13) as well as non-pharmacologic treatments such as skin to skin contact, breastfeeding, rooming in and infant positioning. A previous systematic review and meta-analysis reported that exposure to any opioid during pregnancy was associated with lower birthweight, shorter birth length, smaller head circumference, and increased risk of preterm birth (14). Previous studies have found that preterm birth is associated with lower intelligence (15) and poor academic attainment in early childhood (16–19). Smaller brain volume, which is associated with smaller head circumference among prenatally opioid exposed children (14, 20), is also associated with poor academic achievement (7, 21). Therefore, opioid use during pregnancy has the potential to affect these functions over the life span.

A recent systematic review and meta-analysis found that cognitive and motor skills were lower among children prenatally exposed to opioids (7). That study found significant statistical heterogeneity present across the included studies but did not comprehensively assess the sources of such heterogeneity.

Heterogeneity in the studies included in systematic reviews is often cited as a main reason for not conducting meta-analysis (22, 23). There are different sources of heterogeneity. Clinical heterogeneity is used to refer to the variabilities in study participants, interventions(exposures) and outcomes. Methodological heterogeneity refers to the differences in study design, measurement tools for outcomes and risk of bias. Statistical heterogeneity describes the variabilities in the observed effect estimates, which could be influenced by methodological or clinical heterogeneity. Statistical heterogeneity is manifested by observing more differences between the effect estimates than one would expect to observe by random chance alone (24). In such circumstances, it is important to explore the sources of such heterogeneity and interpret the observed pooled effect estimates accounting for the sources of heterogeneity (22, 23). A comprehensive assessment of heterogeneity in the literature related to prenatal opioid exposure (POE) and neurodevelopmental outcomes will inform to tailor the analysis to account for these variabilities in individual studies and offer improved interpretations of pooled effect estimates.

The aims of this review are to (a) explore the sources of heterogeneity among studies that investigate the neurodevelopmental outcomes of POE children and (b) to estimate the relationship between POE and neurodevelopmental outcomes. We further aimed to investigate and describe the differences in cognitive and motor skills as well as behavior of children prenatally exposed to opioids. We hypothesized that the children with prenatal opioid exposure would have poorer neurodevelopmental outcomes than those without prenatal opioid exposure.

## 2. Material and methods

We used the Covidence systematic review platform to facilitate study selection, data extraction and assessment of the quality of the studies. The study was registered in Prospero (CRD42020153532). This systematic review followed the “Preferred Reporting Items for Systematic Review and Meta-Analyses” (PRISMA) (Supplementary Table S1).

### 2.1. Data sources and selection

We searched four databases through October 17th, 2019: PubMed, Embase, PsycInfo and the Web of Science based on a search string that was designed in consultation with a medical research librarian to fit our inclusion criteria (Supplementary Table S2). We conducted an additional search on May 21st, 2022, limited to studies that were published from 2019 to 2022 to ensure that recently published studies were also included in our review. Two authors (AAB and RIG) independently screened the titles and abstracts, and subsequently the full texts of all identified studies for eligibility, resulting in 79 studies to be included in the systematic review. Conflicts were resolved in discussion among the two investigators in consultation with the senior author (PFL).

We included cohort and case-control studies in our systematic review. Inclusion criteria were studies which had undergone peer-review and were published with full text in English and included human participants and neurodevelopmental outcomes. We did not place any restriction criteria on the publication date or location of the studies. Studies were required to have an exposed group of pregnant women who used opioids during the prenatal period and a comparison group of opioid-unexposed pregnant women. Studies that used the general population as an unexposed group were considered in this review despite the likely presence of individual cases of exposure to opioids in the general population. However, we assumed a low prevalence of prenatal opioid use in the general population. Systematic and narrative reviews, case studies and case series and conference abstracts were not included in this review. We did not include the studies which evaluated fetal alcohol syndrome. We also excluded the studies with no prenatally opioid unexposed comparison groups.

### 2.2. Quality assessment

Two authors (AAB and RIG) independently assessed the quality of each study using the Newcastle Ottawa Quality Assessment Scale for non-randomized studies (Supplementary Table S3) (25). We resolved the conflicts through discussion and in consultation with the senior author (PFL).

### 2.3. Data extraction

We edited the default data extraction template in “Covidence” to include relevant study data and compared results of the first twenty studies to reach consensus. Two authors (AAB and RIG) continued to extract relevant information from included studies. We discussed the discrepancies as a team to reach consensus when identified by the data extraction sheet. We extracted the following data from each study: study design and population data, inclusion and exclusion criteria, opioid exposed and unexposed group differences. We also abstracted the maternal age range and origin (e.g., clinic, hospital) of the study population.

### 2.4. Exposure

We extracted the following information to ascertain exposure status: opioid assessment method (biomarker, maternal self-report, hospital records, NOWS as marker for exposure), time of assessment, type and the name of the opioid(s) measured and information on co-exposure to other substances. We categorized opioids, as Medication used for Opioid Use Disorder (MOUD), “prescribed for medical use” (usually by a health professional) or “non-medical use” according to prespecified criteria (Supplementary Table S4). Non-opioid substances were classified as “prescribed” medications, “legal” and “illegal” or “other non-opioid” substances (Supplementary Table S5).

### 2.5. Outcomes

We extracted neurodevelopmental outcomes, the age of child at assessment and the information regarding the person who performed the assessment (e.g., teacher, parent, physician). Extracted neurodevelopmental outcomes were grouped into three categories: cognitive development, behavioral and attention related problems and attention deficit hyperactivity disorder (ADHD), and motor development. Neurodevelopmental outcomes assessment age was classified according to stages of human development suggested by Lesser and Pope (26): (a) infancy and toddlerhood (≤18 months old), (b) toddlerhood and early childhood (18 months– 6 years old), (c) middle childhood (7–12 years old), and (d) adolescence (>12 years old).

All available continuous and dichotomous outcomes were extracted from each study for inclusion in the systematic review. Due to a very high possibility of heterogeneity among studies; large variation among the tests used to assess neurodevelopment; and low number of studies available for analysis for each type of test, a meta-analysis was not conducted. Whenever studies were conducted on the same cohort, the findings of the study with larger sample size with the given age was extracted. If multiple studies assessed neurodevelopment in a single cohort in a given age with more than one instrument, we extracted and reported all the relevant neurodevelopmental outcomes.

### 2.6. Synthesis

We synthesized our findings regarding heterogeneity into two categories of clinical and methodological heterogeneity. We used tables and figures to investigate and demonstrate the clinical and methodological heterogeneity. To synthesize the findings regarding neurodevelopment we summarized the findings related to cognition, behavior and motor skills comparing the POE populations to their unexposed peers within age groups. We used the vote counting method (22) to subsequently synthesize and summarize the findings based on direction of the observed neurodevelopmental outcomes in POE children compared to their unexposed peers. We estimated the proportion of studies where adverse neurodevelopmental outcomes were reported(success) in each domain of neurodevelopment and within each age group (total number of trials). We subsequently used Wilson's methods to calculate the confidence intervals for the binomial proportion (22, 27).

## 3. Results

### 3.1. Study characteristics

**Figure 1:**
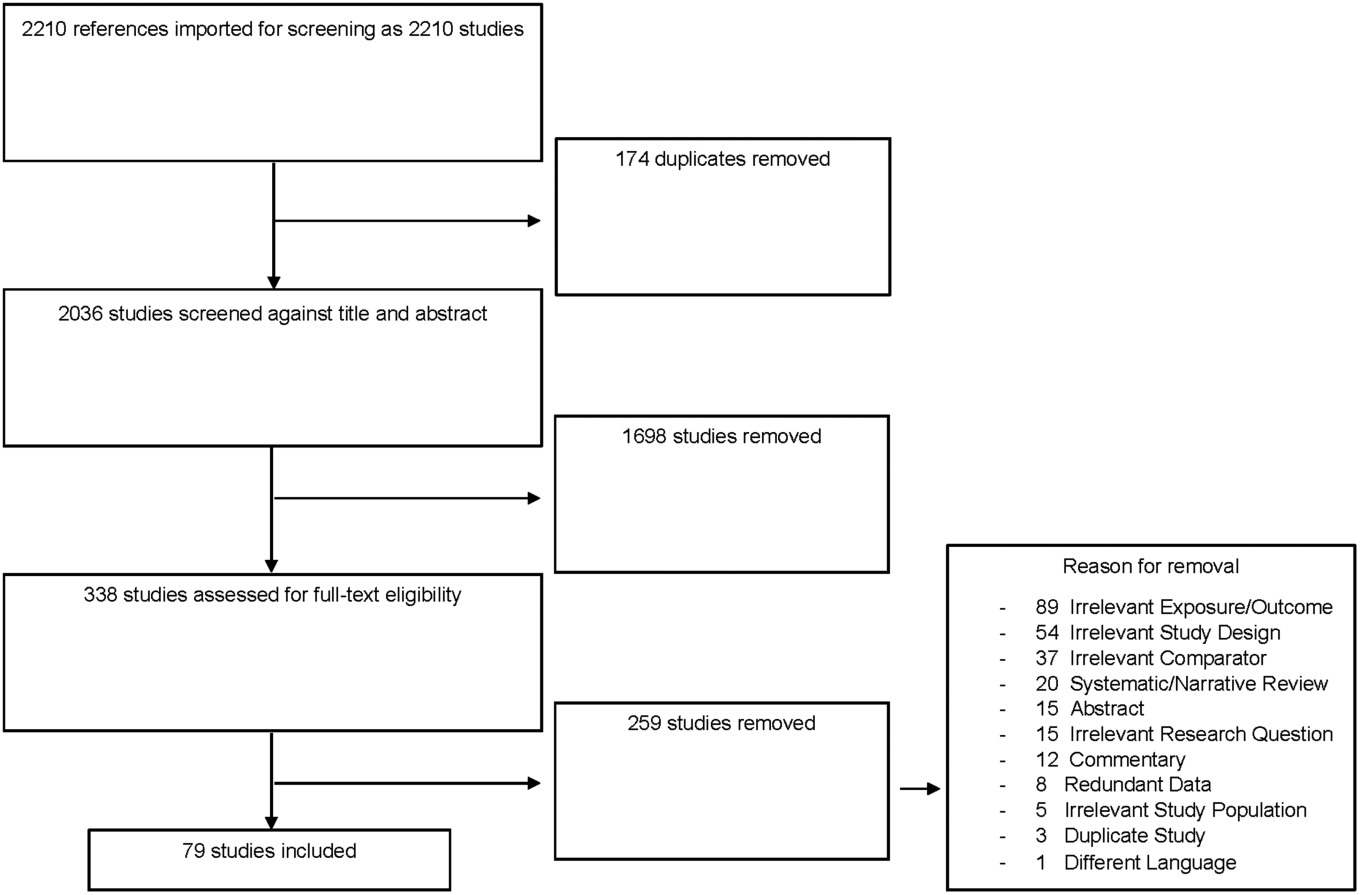
Flowchart of included studies.

The initial literature search yielded 2,210 individual publications. We included 25 additional publications through examination of the reference lists of included studies and previous systematic reviews. After applying our exclusion criteria, final data extraction was conducted on 79 full-text studies (Figure 1).

### 3.2. Heterogeneity

#### 3.2.1. Clinical sources of heterogeneity

Some included studies had several exposed or unexposed groups due to stratification of exposed or unexposed groups based on socioeconomic factors or type of opioids (data not shown). The exposed groups were identified from different settings including “Opioid Centre/Drug Programs” (30 studies), “Hospital/University” (37 studies), “Registry linkage” (8 studies) and “Other/Not specified” (4 studies) (Supplementary Table S6). Additionally, the studies published from 1980 to 1990 mainly assessed exposure to heroin and methadone maintenance therapy. Codeine, oxycodone, and hydrocodone use (prescribed by health professionals or for non-medical use) were rarely assessed among the included studies, regardless of the decade. Buprenorphine replacement therapy was mainly assessed in the studies published from 2011 to 2022 (Supplementary Figure S1).

Most children evaluated for neurodevelopmental outcomes were exposed to more than one class of opioids. Participants of 45 studies reported non-medical (illegal) opioid use, or other types of opioid use, while only four studies included participants that used non-medical opioids exclusively. Over half of the studies reported participants receiving MOUD (60/79) (Supplementary Table S6). Twenty-eight (*n* = 28) studies did not specify partially or fully the opioid drugs name that were used by the exposed groups.

Exposure was assessed at six different time points: before pregnancy, first trimester, second trimester, third trimester, time of delivery and after birth. Thirty-six studies (*n* = 36) assessed the exposure in at least one trimester; the majority of included studies assessed the exposure during delivery or after birth (54/79). Most studies (69/79) did not report stratified results based on exposure window throughout pregnancy in relation to neurodevelopmental outcomes. Of the ten that did, nine studies reported exposure in all trimesters and five specifically assessed opioid use before pregnancy and the neurodevelopmental outcomes (Supplementary Table S6).

Most studies used more than one measure to ascertain perinatal opioid exposure (34/79). Opioid exposure was assessed by self-report (41/79), biological sample (detection of opioids in blood or urine) (36/79), or from hospital records (38/79). Neonatal Abstinence Syndrome/Neonatal Opioid Withdrawal Syndrome (NAS/NOWS) as an indicator for perinatal opioid exposure was used in a few studies (5/79) (Supplementary Table S6).

Most of the exposed and non-exposed children were also exposed to multiple non-opioid substances during pregnancy (e.g., amphetamines, SSRI anti-depressants, barbiturates, benzodiazepines, alcohol, tobacco). The exposed children in 52 studies and unexposed children in 13 studies were also exposed to illegal substances or other substances not including alcohol or tobacco in prenatal period (e.g., cocaine, amphetamines). Co-exposure resulting from medical use of substances such as amphetamines and barbiturates was reported among exposed groups of 39 studies and unexposed groups of nine studies. The exposed children in 51 studies and unexposed children in 37 studies were also exposed to alcohol and/or tobacco. As cannabis is legal both for medical use and adult recreational use in some countries (e.g., Canada, Uruguay) and states in United States and illegal in others, we classified it with other non-opioid substances. The POE groups in 49 studies and unexposed comparison groups in 16 studies were also exposed prenatally to cannabis or other unspecified non-opioid substance.

#### 3.2.2. Methodological sources of heterogeneity

The included studies did not vary significantly by study design. Majority of the included studies were prospective cohort studies (*n* = 52), 25 were retrospective cohort studies, and two were case-control studies.

Nonetheless, we found significant variability regarding the risk of bias among the included studies. We rated 29 studies as “good quality”, 16 studies as “fair quality” and 34 studies as “poor quality” based on Newcastle Ottawa Quality Assessment Tool. The majority of poor studies did not take approaches in the study design or statistical adjustment to ensure comparability between the exposed and unexposed study groups. About half the poor studies had more than 20% loss to follow-up rates among the study populations (Supplementary Table S6).

**Figure 2:**
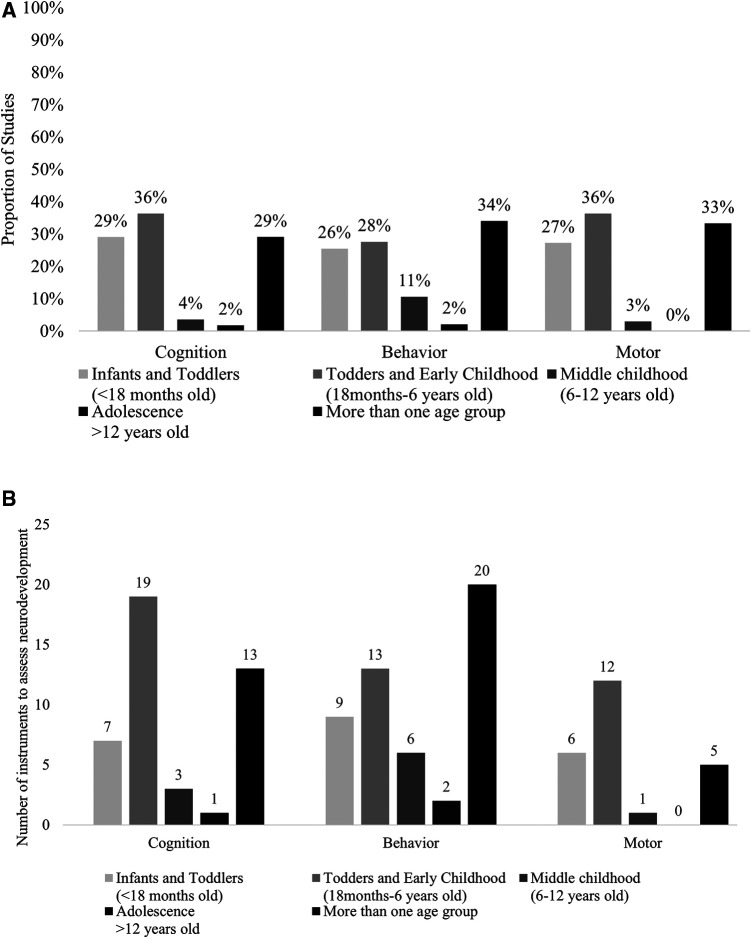
(**A**) Proportion of studies investigating each domain of neurodevelopment by age group. (**B**) Number of instruments investigating each domain of neurodevelopment by age group.

Neurodevelopmental outcomes assessment also varied in different studies. Infants and children were assessed in different ages. A significant proportion of the studies assessed neurodevelopmental outcomes in more than one age group (Figure 2A). Cognitive and motor skills were both assessed mostly among toddlers and early childhood (36%). Thirty-three percent (33%) of the studies assessed behavioral outcomes in more than one age group. Moreover, various instruments were used to assess the same domain of neurodevelopment in different ages between and within the included studies (Figure 2B), contributing to heterogeneity in outcome assessment. For example, cognitive development and its subdomains was assessed using 19 different instruments among toddlers and in early childhood.

#### 3.2.3. Statistical sources of heterogeneity

Although various effect estimates were reported, majority of the studies used t-tests or one-way ANOVA to compare POE children to unexposed peers. The other statistical tests included generalized linear models (e.g., mixed models, linear regression, logistic regression), Wilcoxon rank sum test and chi-squared tests. Numerous studies used matching or statistical adjustment to reduce confounding. Nonetheless, there was a considerable variation in the sociodemographic characteristics used for statistical adjustment or matching (Supplementary Table S6). Due to substantial variance in statistical tests to obtain effect estimates, the specific subdomains of cognition, behavior and motor skills assessed, and other sources of clinical and methodological heterogeneity, we did not calculate pooled measures of effect estimates and measures of statistical heterogeneity.

### 3.3. Neurodevelopmental outcomes

#### 3.3.1. Infants and toddlers (<18 months of age)

##### 3.3.1.1. Cognitive development

**Figure 3:**
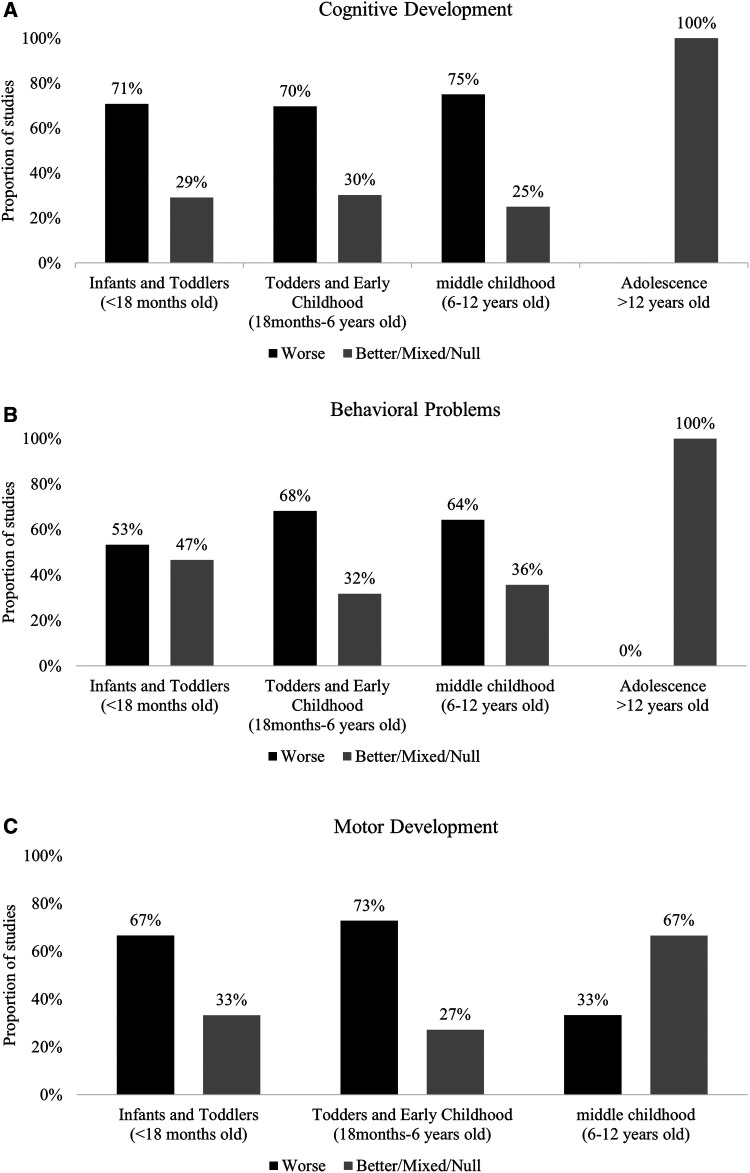
(**A**) Summary of findings in cognition domain by age group. (**B**) Summary of findings in behavior domain by age group. (**C**) Summary of findings in motor development domain by age group.

Cognition was assessed in 24 studies in this age group (29, 30, 32–34, 36, 44, 48–51, 67, 69, 73, 75, 84, 88, 91, 94, 95, 97, 99, 105). The most common test was the Bayley Scale for Infant Development (BSID) (I–III). We found that 71% (95%CI: 49%, 87%, *p* = 0.06) of the studies reported lower scores in various subdomains of cognitive skills among POE infants and toddlers compared to unexposed in this age group (Figure 3A, Supplementary Table S7).

##### 3.3.1.2. Behavioral problems

Fifteen (*n* = 15) studies assessed behavioral problems (30, 31, 33, 44, 48, 55, 60, 61, 65, 69, 84, 93, 98, 105, 106). The most common instruments used were Brazelton Neonatal Behavioral Assessment Scale (BNBAS), BSID-II Behavioral Rating Scale (BRS), BSID III Social-emotional Scale Behavior Rating Inventory. NICU Network Neurobehavioral Scale (NNNS) was used to assess behaviors such as crying, signs of stress and reflexes. In many studies, the children's behavior was assessed longitudinally (28, 44, 46, 55, 69, 76–78, 92, 99, 100). In 53% (95%CI: 26%, 78%, *p* = 1.00) of studies POE infants and toddlers demonstrated unfavorable behaviors compared to unexposed infants and toddlers (Figure 3B, Supplementary Table S8).

##### 3.3.1.3. Motor development

Motor outcomes were assessed in 18 studies in this age group (30–34, 36, 44, 45, 48, 49, 66, 69, 70, 81, 94, 95, 99, 105). Motor development was mainly assessed at birth, among infants and toddlers (3–4 days to 3 years old). POE Infants and toddlers <18 months old in the majority of the included studies [67%, 95%CI: 41%, 87%, *p* = 0.66] had lower scores on different measures of fine and gross motor skills compared to their unexposed peers (Figure 3C, Supplementary Table S9). The test most commonly used (25/34 studies) was the Bayley Scale for Infant Development (30, 32, 33, 36, 44, 48, 49, 66, 69, 70, 75, 81, 94, 95, 99).

#### 3.3.2. Toddlers and early childhood (18 months−<6 years old)

##### 3.3.2.1. Cognitive development

Cognition development was assessed in 32 studies among toddlers and in early childhood (31, 35, 36, 39, 42, 44, 46, 51–55, 59, 63, 68, 69, 71, 73, 75, 79–83, 88, 90, 95, 99, 100, 102–104). The most common test was the Bayley Scale for Infant Development (BSID) (I–III). Children older than 3 years old were assessed by instruments such as McCarthy Scales of Children's Abilities and Wechsler Intelligence Scale for Children. These instruments have various subscales and are used to evaluate higher cognitive skills such as language development, memory, and perception. We found that 72% (95%CI: 53%, 86%, *p* = 0.02) of the studies reported lower scores in various subdomains of cognitive development among POE toddlers and in early childhood compared to unexposed in this age group (Figure 3A, Supplementary Table S7).

##### 3.3.2.2. Behavioral problems

Behavioral problems were assessed in 22 studies (28, 35, 39, 42–44, 46, 47, 54–56, 59, 62, 63, 68, 69, 83, 84, 86, 92, 99, 100) in this age group. The most common instrument used to assess behavior was the Child Behavior Checklist (CBCL) for children older than 3 years old. In 15 studies [68%, 95%CI: 45%, 86%, *p* = 0.13], POE children demonstrated more internalizing and externalizing behavior and attention problems or had higher scores on ADHD scales (Figure 3B, Supplementary Table S8).

##### 3.3.2.3. Motor development

Motor Development was assessed in 22 studies (28, 31, 36, 43, 44, 46, 49, 53, 54, 59, 62, 63, 68–70, 75, 79–81, 95). Most studies used Bayley Scale for Infant Development (BSID) (I–III) to assess motor development among infants and toddlers until 3 years of age. Most studies [*n* = 16, 72%, 95%CI (50%, 89%)] reported lower motor development scores among POE children compared to their unexposed peers (Figure 3C, Supplementary Table S9).

#### 3.3.3. Middle childhood (6−<12 years old)

##### 3.3.3.1. Cognitive development

Cognition development was assessed in *n* = 8 studies in middle childhood (31, 37, 38, 58, 73, 74, 76, 101). Most studies used Wechsler Intelligence Scales for Children to assess cognition. Majority [*n* = 6, 75%, (95%CI: 35%, 97%, *p* = 0.14)] of the studies found that POE children in middle childhood had lower scores in various subdomains of cognitive development compared to unexposed control groups (Figure 3A, Supplementary Table S7).

##### 3.3.3.2. Behavioral problems

Behavioral problems were assessed in 14 studies in this age group (30, 37, 38, 40, 47, 57, 71, 72, 76, 78, 85, 87, 96, 98). The most common instrument (*n* = 6) used to assess behavior was the Child Behavior Checklist (CBCL) in middle childhood. Three studies specifically assessed ADHD. Overall, most studies reported that the POE children in middle childhood tended to have more problems related to behavior compared to unexposed controls (64%, 95%CI: 35%, 87%, *p* = 0.42) (Figure 3B, Supplementary Table S8).

##### 3.3.3.3. Motor development

Motor Development was assessed in 3 studies (28, 37, 41). Two studies reported higher scores on tests to assess motor development among POE children compared to unexposed (Figure 3C, Supplementary Table S9).

#### 3.3.4. Adolescence (≥12 years old)

One study explored cognitive skills and behavioral outcomes among adolescents. Ornoy et al. (2010) (77) found no difference in cognitive development and behavioral outcomes among the POE adolescents compared to the unexposed control group (Figures 3A, B, Supplementary Tables S7, S8).

## 4. Discussion

This paper examined 79 studies that compared children with in-utero opioid exposure to those unexposed to explore neurodevelopmental outcomes. Our findings build upon the previous review (7) by exploring the behavior among POE children compared to their prenatally unexposed peers. Motor outcomes in middle childhood were not discussed in the previous review. Furthermore, we also thoroughly assessed different sources of heterogeneity that was also present in the previous review (7).We found that exposed infants and toddlers performed worse on tests of overall cognitive and motor skills and on behavior assessments compared to those who were unexposed. Results were less consistent for older children particularly for the motor skills in middle childhood.

Nonetheless, results from included studies were extremely heterogenous. Numerous factors could explain this heterogeneity. Firstly, this systematic review had comprehensive inclusion criteria and included all peer-reviewed studies published in English with no restriction on date. Thus, the studies included in this review represented different cohorts and populations. Furthermore, different cohorts studied in the included papers in this review have experienced unique circumstances and conditions such as sociodemographic changes, and patterns of exposure (107). These changes were manifested in the type of exposures assessed in the studies conducted in different decades. The patterns and types of opioid use have changed from the 1970s through 2022 (108). There were differences in type of opioids assessed among the included studies based on the date the studies were published. The studies published prior to 2010 primarily focused on non-medical opioid use such as heroin and MOUD (mainly methadone), while the studies published after 2001, also focused on prescribed opioids by healthcare professional such as codeine and other forms of MOUD (i.e., Buprenorphine). These differences have likely contributed to clinical heterogeneity as manifestations based on the type of opioid are likely different (Supplementary Figure S1).

Included studies differed by setting, population type and size, exposure assessment time, type and time-period of opioid substance and co-exposure use. Opioid exposed and unexposed mother infant pairs were selected at different times before, during, or after pregnancy to participate in the studies. Exposure window was specified only in few studies. Thus, it was challenging to identify critical window of perinatal exposure to opioids and whether the magnitude of neurodevelopmental outcomes would differ based on the exposure period.

Different methods to assess exposure is a significant source of clinical heterogeneity. Several studies reported assessment of exposure by maternal self-report or from medical records of the pregnant women. Many studies used more than one method to assess exposure. We also noted different types of opioids as exposure including MOUD, non-medical and opioids prescribed. Use of different assessment methods and types of opioids have likely increased the heterogeneity among the studies. Nevertheless, use of biomarkers or hybrid measures of exposure assessment can reduce the possibility of social desirability bias or recall bias that can arise when the exposure is assessed based on maternal self-report alone. Only 45% (36/79) of the included studies assessed exposure using biomarkers.

Various studies examined the neurodevelopment of children at different ages. In the studies included in our review, infants, toddlers, and young children were evaluated using as many as 23 distinct instruments. The majority of these instruments were developed to assess the same underlying construct in similar age groups. Nonetheless, different instruments may not evaluate many aspects of early neurodevelopment precisely or similarly. Due to the limited cognitive, motor, and socioemotional skills development in infants and young children, assessment of these skills is challenging in this age group (109). A newborn, for example, has restricted communication and motor abilities, such as weak head control and crying. As infants mature, they reach developmental milestones (26) such as sitting, holding their head, attention and remembering throughout their first year of life. Language development occurs between 12 and 18 months of life (26). Consequently, despite the availability of reliable and valid instruments for assessing neurodevelopmental skills in a certain age group, use of age-specific instruments has contributed to methodological heterogeneity. Furthermore, the domains that can be assessed depend on the child's age. For example, verbal memory cannot be assessed among infants using Wechsler Intelligence Scale for Children (WISC) instrument. Instead, BSID tests are designed to assess the neurodevelopment of very young aged children (110–112). Moreover, the instruments measure the same underlying domain differently. For example, cognitive development includes subdomains such as language development, intelligence, memory, and perception. Each of these subscales is measured in different ways by earlier versions of the BSID (111), WISC (113), MSCA. (114) and the versions that were developed later. The scores of specific subscales in neurodevelopmental tests administered at early ages are correlated with subscales from different tests measuring similar constructs at later ages (115). However, the magnitude of the correlations, at least for infant tests vs. tests at older ages, are relatively small. This indicates some instruments such as BSID might have poor predictive value for neurodevelopmental outcomes in later childhood (116–118).

Although most of the included studies were longitudinal prospective cohorts, we found significant sources of methodological heterogeneity as the studies differed in quality based on our quality assessment scale. These differences included: the recruitment of exposed and unexposed populations; representativeness of study population; modes of independent assessment of outcomes (e.g., assessment of neurodevelopment in children blinded to opioid exposure assessment); statistical adjustment or other method to reduce confounding (e.g., matching); and whether retention is independent of exposure and outcome status. For example, the lost to follow-up was significantly higher among the exposed groups compared to unexposed groups in some studies (49, 88).

All these factors resulted in high clinical and methodological heterogeneity (119, 120). Although some statistical methods such as using random effects models and stratifying the studies by age groups at which children were evaluated (7) could minimize the statistical heterogeneity associated with clinical and methodological heterogeneous studies, these methods could not control all the sources of heterogeneity that were present in this review. Pooling the summary estimates from these heterogenous studies could result in erroneous pooled measures and misleading conclusions (119).

Several limitations in the included studies could have influenced the overall findings of our review. A potential limitation of most of the included studies was the absence of comparability between prenatally opioid exposed and unexposed children as these studies did not take any measure such as statistical adjustment or matching to ensure exchangeability. The lack of comparability arises due to inherent differences between the women who misuse opioids with the unexposed comparison groups. Therefore, recruiting comparable unexposed comparison groups in the study design stage is crucial, yet challenging when studying the association between prenatal opioid exposure and neurodevelopmental outcomes. Confounding factors such as social class or school, family and neighborhood could have influenced the results of the studies conducted in different geographic areas in different ways (56, 62, 121). The absence of comparability has also contributed to heterogeneity of the studies. Confounding by the co-exposures could also distort the findings of individual studies and influence our study. As explained earlier the opioid exposed groups were also exposed to various non-opioid substances. Prenatal exposure to some of these substances such as alcohol and cigarette smoke is associated with poor neurodevelopmental outcomes.

Another limitation was the possibility of selection bias due to possible differential loss to follow-up or non-response from the mothers of infants with POE. Finally, possibility of type-II errors among the included studies could not be disregarded due to absence of clear sampling strategy, sample size calculation or *post hoc* power calculation.

Besides the limitations in the included studies, several other issues have likely influenced our review. First, we did not include grey and non-peer reviewed literature. The studies included in our review were predominantly conducted in North America and Europe. No studies were included from the regions such as South-West Asia/Near-Middle east or published in non-English languages in our review. Considering the high prevalence of opioid use (122), and variability in access to MOUD and prenatal care in the latter regions, inclusion of studies conducted on this topic would increase both clinical and methodological heterogeneity in our review. Second, our review does not provide a pooled effect estimate. Using methods such as subgroup analyses was not possible due to numerous methods of exposure and outcome measurement. Thus, this review did not include a meta-analysis to pool the summary estimates, rather it is highlighting the current state of literature regarding the association between POE and neurodevelopmental outcomes. It further identifies the sources of heterogeneity between the studies, with the aim of helping to standardize methods in future studies. Third, we did not extract the study data regarding sex-specific associations on neurodevelopmental outcomes. Nonetheless, most of the studies did not stratify the neurodevelopmental outcomes by sex. It is possible that the harmful effects of POE are different for girls and boys. Such differences were observed in several studies of environmental exposures and child neurodevelopment (123, 124). We did not plan to extract information regarding psychometric properties of the instruments used to assess neurodevelopment; however, this information was not available in many studies. Moreover, instruments with different validity and reliabilities could further contribute to the methodological heterogeneity. Finally, the absence of information regarding the prenatal exposure assessment period during pregnancy, or the name and dose of the opioid drug used by pregnant women in some studies, complicated our conclusions about sources of heterogeneity of the included studies.

Our study also had several strengths. First, we comprehensively included studies in terms of the type of opioid used, study design, and date of publication. Second, despite differences in exposed and unexposed groups, the effect of opioids on all the domains of neurodevelopmental outcomes was consistent particularly among infants and toddlers. Third, the choice of comparison group to be unexposed to any opioids provided a contrast to compare the neurodevelopmental outcomes in exposed and unexposed neonates. Fourth, the comprehensive assessment of sources of heterogeneity could be used to make the future studies more homogenous. This goal can be accomplished by using validated instruments to assess neurodevelopment and thorough exposure assessment and specifying the period of exposure assessment.

## 5. Conclusions

Our study demonstrated large clinical and methodological heterogeneity among studies that have assessed the impact of in-utero opioid exposure on neurodevelopmental outcomes. The sources of heterogeneity were mainly in measurements of exposure to opioids and neurodevelopmental outcomes. The studies also varied in terms of the approaches taken to ensure comparability among the exposed and unexposed populations. The children who were exposed to opioids in the perinatal period tended to have lower scores on tests of cognitive and motor skills and demonstrated more problems regarding internalizing and externalizing behavior as well as attention related problems compared to unexposed children. Our finding regarding substantial prenatal exposure to other non-opioid substances informs the necessity of studies investigating the additive effect of opioids with co-exposures on children neurodevelopment. Future studies should improve the comparability of groups exposed and unexposed to opioids by design or statistical adjustment. Furthermore, the studies should clearly specify the prenatal exposure period that are assessed in relation to neurodevelopmental outcomes, and possibilities of interactions with other non-opioid substances.

## Data Availability

The original contributions presented in the study are included in the article/**Supplementary Material**, further inquiries can be directed to the corresponding author.
