# Supplementary material for: Prenatal exposure to opioids and neurodevelopment in infancy and childhood: A systematic review
Source: Front Pediatr. 2023 Feb 21;11:1071889. doi: 10.3389/fped.2023.1071889 (PMC9989202; doi:10.3389/fped.2023.1071889)
Supplement: Supplementary file 1 [file Datasheet1.docx]

Supplementary Material

**Contents**

[1.1 Supplemental Tables 2](#_Toc126335212)

[Supplemental Table1: PRISMA 2020 Main Checklist 2](#_Toc126335213)

[Supplemental Table 2: Search Strings for Systematic Review 8](#_Toc126335214)

[Supplemental Table 3: New Castle Ottawa Quality Assessment Scale 10](#_Toc126335215)

[Supplemental Table 4 Opioids classification 15](#_Toc126335216)

[Supplemental Table 5: Co-exposures classification 16](#_Toc126335217)

[Supplemental Table 6: Characteristics of the included studies in the systematic review 17](#_Toc126335218)

[Supplemental Table 7: Studies Investigating Prenatal Exposure to Opioids and Cognitive Development 35](#_Toc126335219)

[Supplemental Table 8: Studies Investigating Prenatal Exposure to Opioids And Behavioral Development 69](#_Toc126335220)

[Supplemental Table 9: Studies Investigating Prenatal Exposure to Opioids and Motor Development 96](#_Toc126335221)

[1.2 Supplemental Figures 108](#_Toc126335222)

[Supplemental Figure 1. Proportion of studies assessing different types of prenatal exposure to opioids among all the included studies published from 1970-2020 based on subject recruitment period 109](#_Toc126335223)

## Supplemental Tables

### Supplemental Table1: PRISMA 2020 Main Checklist

| Topic | No. | Item | Location where item is reported |
| --- | --- | --- | --- |
| TITLE |  |  |  |
| Title | 1 | Identify the report as a systematic review. | 1 |
| ABSTRACT |  |  |  |
| Abstract | 2 | See the PRISMA 2020 for Abstracts checklist |  |
| INTRODUCTION |  |  |  |
| Rationale | 3 | Describe the rationale for the review in the context of existing knowledge. | 6 |
| Objectives | 4 | Provide an explicit statement of the objective(s) or question(s) the review addresses. | 6 |
| METHODS |  |  |  |
| Eligibility criteria | 5 | Specify the inclusion and exclusion criteria for the review and how studies were grouped for the syntheses. | 7 |
| Information sources | 6 | Specify all databases, registers, websites, organisations, reference lists and other sources searched or consulted to identify studies. Specify the date when each source was last searched or consulted. | 7 |
| Search strategy | 7 | Present the full search strategies for all databases, registers and websites, including any filters and limits used. | eTable 2 |
| Selection process | 8 | Specify the methods used to decide whether a study met the inclusion criteria of the review, including how many reviewers screened each record and each report retrieved, whether they worked independently, and if applicable, details of automation tools used in the process. | 7 |
| Data collection process | 9 | Specify the methods used to collect data from reports, including how many reviewers collected data from each report, whether they worked independently, any processes for obtaining or confirming data from study investigators, and if applicable, details of automation tools used in the process. | 7 |
| Data items | 10a | List and define all outcomes for which data were sought. Specify whether all results that were compatible with each outcome domain in each study were sought (e.g. for all measures, time points, analyses), and if not, the methods used to decide which results to collect. | 8-9 |
|  | 10b | List and define all other variables for which data were sought (e.g. participant and intervention characteristics, funding sources). Describe any assumptions made about any missing or unclear information. | 8-9 |
| Study risk of bias assessment | 11 | Specify the methods used to assess risk of bias in the included studies, including details of the tool(s) used, how many reviewers assessed each study and whether they worked independently, and if applicable, details of automation tools used in the process. | Page 8, Table 1 |
| Effect measures | 12 | Specify for each outcome the effect measure(s) (e.g. risk ratio, mean difference) used in the synthesis or presentation of results. | Appendix 2 |
| Synthesis methods | 13a | Describe the processes used to decide which studies were eligible for each synthesis (e.g. tabulating the study intervention characteristics and comparing against the planned groups for each synthesis (item 5)). | 8-9 |
|  | 13b | Describe any methods required to prepare the data for presentation or synthesis, such as handling of missing summary statistics, or data conversions. | 9 |
|  | 13c | Describe any methods used to tabulate or visually display results of individual studies and syntheses. | N/A |
|  | 13d | Describe any methods used to synthesize results and provide a rationale for the choice(s). If meta-analysis was performed, describe the model(s), method(s) to identify the presence and extent of statistical heterogeneity, and software package(s) used. | 9 |
|  | 13e | Describe any methods used to explore possible causes of heterogeneity among study results (e.g. subgroup analysis, meta-regression). | 14-16 |
|  | 13f | Describe any sensitivity analyses conducted to assess robustness of the synthesized results. | N/A |
| Reporting bias assessment | 14 | Describe any methods used to assess risk of bias due to missing results in a synthesis (arising from reporting biases). | 8 |
| Certainty assessment | 15 | Describe any methods used to assess certainty (or confidence) in the body of evidence for an outcome. | N/A |
| RESULTS |  |  |  |
| Study selection | 16a | Describe the results of the search and selection process, from the number of records identified in the search to the number of studies included in the review, ideally using a flow diagram. | 9, Figure1 |
|  | 16b | Cite studies that might appear to meet the inclusion criteria, but which were excluded, and explain why they were excluded. | N/A |
| Study characteristics | 17 | Cite each included study and present its characteristics. | Table 1 |
| Risk of bias in studies | 18 | Present assessments of risk of bias for each included study. | Table 1 |
| Results of individual studies | 19 | For all outcomes, present, for each study: (a) summary statistics for each group (where appropriate) and (b) an effect estimate and its precision (e.g. confidence/credible interval), ideally using structured tables or plots. | 12-13  Appendix 2 |
| Results of syntheses | 20a | For each synthesis, briefly summarise the characteristics and risk of bias among contributing studies. | N/A |
|  | 20b | Present results of all statistical syntheses conducted. If meta-analysis was done, present for each the summary estimate and its precision (e.g. confidence/credible interval) and measures of statistical heterogeneity. If comparing groups, describe the direction of the effect. | N/A |
|  | 20c | Present results of all investigations of possible causes of heterogeneity among study results. | 10-11 |
|  | 20d | Present results of all sensitivity analyses conducted to assess the robustness of the synthesized results. | N/A |
| Reporting biases | 21 | Present assessments of risk of bias due to missing results (arising from reporting biases) for each synthesis assessed. | N/A |
| Certainty of evidence | 22 | Present assessments of certainty (or confidence) in the body of evidence for each outcome assessed. | N/A |
| DISCUSSION |  |  |  |
| Discussion | 23a | Provide a general interpretation of the results in the context of other evidence. | 16 |
|  | 23b | Discuss any limitations of the evidence included in the review. | 17 |
|  | 23c | Discuss any limitations of the review processes used. | 17-18 |
|  | 23d | Discuss implications of the results for practice, policy, and future research. | 19 |
| OTHER INFORMATION |  |  |  |
| Registration and protocol | 24a | Provide registration information for the review, including register name and registration number, or state that the review was not registered. | 7 |
|  | 24b | Indicate where the review protocol can be accessed, or state that a protocol was not prepared. | 7 |
|  | 24c | Describe and explain any amendments to information provided at registration or in the protocol. | N/A |
| Support | 25 | Describe sources of financial or non-financial support for the review, and the role of the funders or sponsors in the review. | 19 |
| Competing interests | 26 | Declare any competing interests of review authors. | N/A |
| Availability of data, code and other materials | 27 | Report which of the following are publicly available and where they can be found: template data collection forms; data extracted from included studies; data used for all analyses; analytic code; any other materials used in the review. | N/A |

*From:* Page MJ, McKenzie JE, Bossuyt PM, Boutron I, Hoffmann TC, Mulrow CD, et al. The PRISMA 2020 statement: an updated guideline for reporting systematic reviews. MetaArXiv. 2020, September 14. DOI: 10.31222/osf.io/v7gm2. For more information, visit: [www.prisma-statement.org](file:///C:\Users\arinb\AppData\Local\Temp\Rar$DIa0.639\www.prisma-statement.org)

### Supplemental Table 2: Search Strings for Systematic Review

| **Pubmed** | ((((((((((((((((((Psychomotor Performance[MeSH Terms]) OR Neurocognitive Disorders[MeSH Terms]) OR Cognitive Dysfunction[MeSH Terms]) OR Motor Disorders[MeSH Terms]) OR Child Development[MeSH Terms]) OR (growth[MeSH Subheading] AND development[MeSH Subheading])) OR Child Behavior Disorders[MeSH Terms]) OR Developmental Disabilities[MeSH Terms]) OR Neurodevelopmental Disorders[MeSH]) OR adolescent behavior[MeSH Terms]) OR Child Behavior[MeSH Terms]) OR cognit* development) OR motor development) OR academic performance) OR attainment) OR school performance)) AND (((((((((pregnancy[MeSH Terms]) OR gravidity[MeSH Terms]) OR Maternal-Fetal Relations[MeSH Terms]) OR prenatal exposure delayed effects[MeSH Terms]) OR Maternal  Exposure[MeSH Terms]) OR in utero) OR prenatal) OR fetal) OR Neonatal Abstinence Syndrome[MeSH Terms])) AND (((((((((((((((((Opiate*) OR Opioid*) OR addiction, opiate[MeSH Terms]) OR heroin) OR opium) OR Buprenorphine) OR Methadone) OR Oxycodone) OR Hydrocodone) OR Codeine) OR Fentanyl) OR Analgesics, Opioid [Pharmacological Action]) OR Analgesics, Opioid[MeSH Terms]) OR Opioid-related Disorders[MeSH Terms]) OR Heroin Dependence[MeSH Terms]) OR Neonatal Abstinence  Syndrome[MeSH Terms])) NOT Cocaine[MeSH Terms]  Filters: Humans; Clinical Trial, Controlled Clinical Trial, English Abstract, Journal Article, Meta-Analysis, Observational Study, Randomized Controlled Trial, Systematic Reviews; English |
| --- | --- |
| **Embase** | ('psychomotor performance'/exp OR 'disorders of higher cerebral function'/exp OR 'cognitive defect'/exp OR 'motor dysfunction'/exp OR 'child development'/exp OR 'growth, development and aging'/exp OR 'behavior disorder'/exp OR 'developmental delay'/exp OR 'mental disease'/exp OR 'adolescent behavior'/exp OR 'child behavior'/exp OR 'cognitive development'/exp OR 'motor development'/exp OR 'academic achievement'/exp OR 'mental capacity'/exp) AND ('pregnancy'/exp OR  'mother fetus relationship'/exp OR 'prenatal exposure'/exp OR 'maternal exposure'/exp OR 'in utero exposure'/exp OR 'neonatal abstinence syndrome'/exp) AND ('opiate'/exp OR 'opiate addiction'/exp OR 'diamorphine'/exp OR 'buprenorphine'/exp OR 'methadone'/exp OR 'oxycodone'/exp OR 'hydrocodone'/exp OR 'codeine'/exp OR 'fentanyl'/exp OR 'fentanyl derivative'/exp OR 'tramadol'/exp OR 'opiate agonist'/exp OR 'heroin dependence'/exp OR 'morphine addiction'/exp OR 'neonatal abstinence syndrome'/exp) NOT 'fetal alcohol syndrome'/exp NOT 'cocaine'/exp AND ([article]/lim OR [review]/lim) AND [humans]/lim AND [article]/lim AND [english]/lim AND [embase]/lim |
| **Web of Science** | (((((((((((((((((Psychomotor Performance) OR Neurocognitive Disorder) OR Cognitive Dysfunction) OR Motor Disorder) OR Child Development) OR (growth AND development)) OR Child Behavior Disorder) OR Developmental Disability) OR Neurodevelopmental Disorder) OR adolescent behavior) OR Child Behavior) OR cognit* development) OR motor development) OR academic performance) OR attainment) OR school performance)) AND (((((((((pregnancy) OR gravidity) OR Maternal-Fetal Relations) OR prenatal exposure delayed effects) OR Maternal Exposure) OR in utero) OR prenatal) OR fetal) OR Neonatal Abstinence Syndrome)) AND (((((((((((((((((((((((((((((((((((((((((((((((Opiate*) OR Opioid*) OR addiction, opiate) OR Opioid Analgesics) OR Opioid-related Disorders) OR Heroin Dependence) OR Neonatal Abstinence Syndrome) OR Alfentanil) OR Alphaprodine)OR Buprenorphine) OR Butorphanol) OR Codeine) OR Dextromoramide) OR Dextropropoxyphene) OR Dihydromorphine) OR Diphenoxylate) OR Ethylketocyclazocine) OR Ethylmorphine) OR Etorphine) OR Fentanyl) OR Heroin) OR Hydrocodone) OR Hydromorphone) OR Levorphanol) OR Meperidine) OR Meptazinol) OR Methadone) OR Methadyl Acetate) OR Morphine) OR Nalbuphine) OR Opiate Alkaloids) OR Opium) OR Oxycodone) OR Oxymorphone) OR Pentazocine) OR Phenazocine) OR Phenoperidine) OR Pirinitramide) OR Promedol) OR Remifentanil) OR Sufentanil) OR Tapentadol) OR Tilidine) OR Tramadol) OR Pethidine)) NOT Cocaine  Filters: English |
| **PsychInfo** | ((Psychomotor Performance* OR Neurocognitive Disorder* OR Cognitive Dysfunction* OR Motor Disorder* OR Child Development OR (growth AND development) OR Child Behavio?r Disorder* OR Developmental Disabilit* OR Neurodevelopmental Disorder* OR adolescent behavio?r OR Child Behavio?r OR cognit* development OR motor development OR academic performance OR attainment OR school performance) AND (pregnan* OR gravidit* OR Maternal-Fetal Relation* OR prenatal exposure delayed effect* OR Maternal Exposure OR in utero OR prenatal OR fetal OR Neonatal Abstinence Syndrome) AND (Opiate* OR Opioid* OR addiction, opiate OR Opioid Analgesic* OR Opioid-related Disorder* OR Heroin Dependence OR Neonatal Abstinence Syndrome OR Alfentanil OR Alphaprodine OR Buprenorphine OR Butorphanol OR Codeine OR Dextromoramide OR Dextropropoxyphene OR Dihydromorphine OR Diphenoxylate OR Ethylketocyclazocine OR Ethylmorphine OR Etorphine OR Fentanyl OR Heroin OR Hydrocodone OR Hydromorphone OR Levorphanol OR Meperidine OR Meptazinol OR Methadone OR Methadyl Acetate OR Morphine OR Nalbuphine OR Opiate Alkaloids OR Opium OR Oxycodone OR Oxymorphone OR Pentazocine OR Phenazocine OR Phenoperidine OR Pirinitramide OR Promedol OR Remifentanil OR Sufentanil OR Tapentadol OR Tilidine OR Tramadol OR Pethidine) NOT Cocaine)  **Refined by: DOCUMENT TYPES:** ( ARTICLE OR OTHER OR CLINICAL TRIAL OR REVIEW OR ABSTRACT ) AND**Databases:** ( WOS ) AND [excluding]**Databases:** ( MEDLINE )]  Language: English |

### Supplemental Table 3: New Castle Ottawa Quality Assessment Scale

1. **Cohort Studies**

| Item |  | Put star if true |  |
| --- | --- | --- | --- |
| Selection | | | |
| 1- Representativeness of the exposed cohort |  |  |  |
|  | a) truly representative ⁕ |  |  |
|  | b) somewhat representative⁕ |  |  |
|  | c) selected group (no star) |  |  |
|  | d) no description of the derivation of the cohort (no star) |  |  |
| 2- Selection of the non-exposed cohort |  |  |  |
|  | a) drawn from the same community as the exposed cohort⁕ |  |  |
|  | b) drawn from a different source (no star) |  |  |
|  | c) no description of the derivation of the non-exposed cohort (no star) |  |  |
| 3) Ascertainment of exposure |  |  |  |
|  | a) secure record (e.g., surgical records) ⁕ |  |  |
|  | b) structured interview⁕ |  |  |
|  | c) written self-report (no star) |  |  |
|  | d) no description (no star) |  |  |
| 4) Demonstration that outcome of interest was not present at start of study |  |  |  |
|  | a) yes⁕ |  |  |
|  | b) no (no star) |  |  |
| Comparability | | | |
| 1) Comparability of cohorts on the basis of the design or analysis |  |  |  |
|  | a) study controls for _____________ (select the most important factor) ⁕ |  |  |
|  | b) study controls for any additional factor (This criteria could be modified to indicate specific control for a second important factor.) ⁕ (no star) |  |  |
|  | c) Cohorts are not comparable on the basis of the design or analysis controlled for confounders (no star) |  |  |
| Outcome Domain | | | |
| 1) Assessment of outcome |  |  |  |
|  | a) independent blind assessment⁕ |  |  |
|  | b) record linkage⁕ |  |  |
|  | c) self-report (no star) |  |  |
|  | d) no description (no star) |  |  |
| 2) Was follow-up long enough for outcomes to occur |  |  |  |
|  | a) yes (select an adequate follow up period for outcome of interest) ⁕ |  |  |
|  | b) no (no star) |  |  |
| 3) Adequacy of follow up of cohorts |  |  |  |
|  | a) complete follow up - all subjects accounted for⁕ |  |  |
|  | b) subjects lost to follow up unlikely to introduce bias - small number lost - > ____ % (select an adequate %) follow up, or description provided of those lost) ⁕ |  |  |
|  | c) follow up rate < ____% (select an adequate %) and no description of those lost (no star) |  |  |
|  | d) No description (no star) |  |  |

Good quality: 3 or 4 stars in selection domain AND 1 or 2 stars in comparability domain AND 2 or 3 stars in outcome domain Fair quality: 2 stars in selection domain AND 1 or 2 stars in comparability domain AND 2 or 3 stars in outcome domain Poor quality: 0 or 1 star in selection domain OR 0 stars in comparability domain OR 0 or 1 stars in outcome domain

1. **Case Control Studies**

| Item |  | Put star if true |
| --- | --- | --- |
| Selection | | |
| 1) Is the case definition adequate? |  |  |
|  | a) yes, with independent validation⁕ |  |
|  | b) yes, e.g., record linkage or based on self-reports (no star) |  |
|  | c) no description (no star) |  |
| 2) Representativeness of the cases |  |  |
|  | a) consecutive or obviously representative series of cases⁕ |  |
|  | b) potential for selection biases or not stated (no star) |  |
| 3) Selection of controls |  |  |
|  | a) community controls ⁕ |  |
|  | b) hospital records⁕ |  |
|  | d) no description (no star) |  |
| 4) Definition of Controls |  |  |
|  | a) no history of disease (endpoint)⁕ |  |
|  | b) no description of source (no star) |  |
| Comparability | | |
| 1) Comparability of cases and controls on the basis of the design or analysis |  |  |
|  | a) study controls for _______________ (Select the most important factor.) ⁕ |  |
|  | b) study controls for any additional factor (This criteria could be modified to indicate specific control for a second important factor.) ⁕ |  |
| Exposure Domain | | |
|  |  |  |
| 1) Assessment of exposure |  |  |
|  | a) secure record (e.g., surgical records) ⁕ |  |
|  | b) structured interview where blind to case/control status ⁕ |  |
|  | c) interview not blinded to case/control status (no star) |  |
|  | d)written self-report or medical record only (no star) |  |
|  | d) no description (no star) |  |
| 2) Same method of ascertainment for cases and controls |  |  |
|  | a) yes⁕ |  |
|  | b) no |  |
| Non-Response rate |  |  |
|  | a) same rate for both groups⁕ |  |
|  | b) non respondents described |  |
|  | c) rate different and no designation |  |

Good quality: 3 or 4 stars in selection domain AND 1 or 2 stars in comparability domain AND 2 or 3 stars in exposure domain Fair quality: 2 stars in selection domain AND 1 or 2 stars in comparability domain AND 2 or 3 stars in exposure domain Poor quality: 0 or 1 star in selection domain OR 0 stars in comparability domain OR 0 or 1 stars in exposure domain

### Supplemental Table 4 Opioids classification

| **Opioid Class** | **Opioid Name** |
| --- | --- |
| **Non-Medical Opioids** | Heroin |
|  | Morphine |
|  | Fentanyl |
| **Medication for Opioid Use Disorder (MOUD)** | Methadone Maintenance Therapy |
|  | Buprenorphine Therapy |
|  | Other medication for opioid use disorder (other MOUD) |
| **Prescription opioids obtained legally or illegally** | Tramadol |
|  | Oxycodone |
|  | Codeine |
|  | Other legal Opioid |

### Supplemental Table 5: Co-exposures classification

| **Opioid Class** | **Opioid Name** |
| --- | --- |
| **Illegal** | Cocaine |
|  | Methamphetamines |
|  | Phencyclidine |
|  | LSD |
| **Prescribed** | Amphetamines |
|  | SSRI anti depressants |
|  | Barbiturates |
|  | Benzodiazepines |
| **Legal** | Alcohol |
|  | Tobacco |
| **Other** | Cannabis* |
|  | Other unspecified non-opioid drugs |

*Depending on country and state in United States this substance could be considered legal.

### Supplemental Table 6: Characteristics of the included studies in the systematic review

| **Study Identifier** | **Country/**  **Continent** | **Study Design** | **Origin of the Exposed Group** | **Opioid Assessment Method among POE** | **Time of exposure assessment among POE** | **Type of Opioids used in POE group^a^** | **Type of non-Opioid co exposure used in POE group ^b^** | **Was a specific window of exposure assessed?** | **Age group of children at neurodevelopmental outcomes assessment** | **Adjustment covariates/**  **Matching covariates** | **Was loss to Follow Up rate > 20%?** | **Study**  **Quality assessment** | **Total Sample Size (POE sample size)** | **Type of statistical test** (e.g., Cox regression, Descriptive analysis, logistic regression) | **Who assessed the outcome?**  **(Blinded to POE status?)** |
| --- | --- | --- | --- | --- | --- | --- | --- | --- | --- | --- | --- | --- | --- | --- | --- |
| Azuine 2019(62) | USA/North America | Prospective cohort study | Hospital/  University | Maternal Self-Report | Before Pregnancy,  1^st^ Trimester, 2^nd^ Trimester, 3^rd^ Trimester, Time of Delivery | Non-medical, MOUD | Illegal, Legal | No | Infants and Toddlers  (<18 months old)  Toddlers and Early Childhood  (18months-6 years old)  Middle childhood (6-12 years old) | Adjusted for: maternal age, household income,  race/ethnicity, marital status, and maternal education | Yes | Fair | 8.509 (454) | Multinomial logistic regression | Trained research  staff ^c^ |
| Bada 2002(29) | USA/North America | Prospective cohort study | Hospital/  University | Maternal Self-Report, Biological Sample | Time of Delivery | Non-medical, MOUD, Not specified | Illegal, Legal | No | Infants and Toddlers  (<18 months old) | Adjusted for: Clinical site, GA, postnatal age at assessment, biological sex, race, maternal marital status, highest grade of education completed, Medicaid status, prenatal care, sexually transmitted diseases including AIDS/HIV, and use of cannabis, alcohol, and tobacco during pregnancy | Yes | Good | 7442 (100) | Multivariate modelling approach with generalized estimating equations (GEE) | Trained research staff (blind) |
| Bakhireva 2019(30) | USA/North America | Prospective cohort study | Hospital/  University | Maternal Self-Report, clinical record | 1^st^ Trimester, 2^nd^ Trimester, 3^rd^ Trimester, Time of Delivery | Non-medical, MOUD | Illegal, Legal | No | Infants and Toddlers  (<18 months old) | None | Yes | Poor | 78 (42) | T-tests, Linear fixed effects model and multivariable logistic regression for only some outcomes | Trained pediatricians/physicians, pediatric neurologists, psychologists, developmental psychologists or psychometrists (blind) |
| Bauer 2020(52) | USA/North America | Retrospective cohort study | Hospital/  University | Maternal Self-Report, Biological Sample | After Birth | Non-medical, MOUD | Illegal, Legal | No | Infants and Toddlers  (<18 months old)  Toddlers and Early Childhood  (18months-6 years old)  Middle childhood (6-12 years old) | Matched on gestational age, race, and sex within the same institution  Adjusted for: Study site, biological sex, race, birth weight, gestational age, alcohol exposure and smoking during pregnancy, and maternal education | Yes | Good | 90 (45) | T-tests. Mixed effects models adjusted for covariates. | Trained examiner(s) ^d^ |
| Beckwith 2015(31) | USA/North America | Prospective cohort study | Opioid Centre/Drug Program | biological sample, NOWS as indicator | Not specified | Non-medical, MOUD | Not Specified | No | Infants and Toddlers  (<18 months old) | None | Yes | Poor | 1728 (28) | t-tests and chi-squared tests | Trained pediatricians/physicians, pediatric neurologists, psychologists, developmental psychologists or psychometrists |
| Bernstein 1984(32) | USA/North America | Prospective cohort study | Other/Not Specified | Not Specified | Not specified | MOUD | Not Specified | No | Infants and Toddlers  (<18 months old) | None | Yes | Poor | 40 (17) | T-test | Trained examiner(s) (blind) |
| Bunikowski 1998(33) | Germany /Europe | Prospective cohort study | Hospital/  University | Not specified | Before Pregnancy,  1^st^ Trimester, 2^nd^ Trimester, 3^rd^ Trimester, Not specified | Non-medical, MOUD | Non-medical, Legal, Prescribed | No | Infants and Toddlers  (<18 months old) | None | No | Poor | 76 (34) | Wilcoxon Rank Sum Test, Chi squared or Fisher's exact test for categorical data, and the Spearman correlation coefficients. | Trained pediatricians/physicians, pediatric neurologists, psychologists, developmental psychologists or psychometrists |
| Burns 1996(71) | United Kingdom /Europe | Retrospective cohort study | Opioid Centre/  Drug Program | Maternal Self-Report | 1^st^ Trimester, 2^nd^ Trimester, 3^rd^ Trimester, | Non-medical, MOUD | Legal, Illegal | No | Toddlers and Early Childhood  (18months-6 years old)  Middle Childhood (6-12 years old) | Matched on: Child age and SES | No | Good | 43 (23) | Summary descriptive statistics | Author(s) |
| Chasnoff 1986(34) | USA/North America | Prospective cohort study | Hospital/  University | Biological Sample | 1^st^ Trimester, 2^nd^ Trimester | Non-medical, MOUD, Not specified | Not specified | No | Infants and Toddlers  (<18 months old)  Toddlers and Early Childhood  (18months-6 years old) | Adjusted for: race | Yes | Fair | 78 (51) | Analysis of variance (ANOVA) and chi-squared test; | Trained examiner(s)(blind) |
| Davis 1988(94) | USA/North America | Retrospective cohort study | Opioid Centre/Drug Program | Clinical Record | Not specified | Non-medical, MOUD | Not specified | No | Middle Childhood (6-12 years old) | Adjusted for: children age | No | Good | 56 (28) | F-test | Author(s), teacher(s) (blind) |
| deCubas 1993(95) | USA/North America | Retrospective cohort study | Opioid Centre/Drug Program | Clinical Record | After Birth | MOUD | Legal | No | Middle Childhood (6-12 years old) | SES, race, medical background | No | Good | 40 (20) | Hotelling's T-squared and t-tests | Trained examiner(s) |
| Fill 2018(72) | USA/North America | Case-control study | Registry linkage study | NOWS as Indicator | After Birth | Not specified | Legal, Not specified | No | Toddlers and Early Childhood  (18months-6 years old)  Middle Childhood (6-12 years old) | Matched on: biological sex, race and/or ethnicity, Insurance enrollment status, age, and region of residence  Adjusted for: maternal tobacco use during pregnancy, and maternal education status | No | Good | 7256 (1815) | Chi-squared tests, conditional multivariable logistic regression | Not specified |
| Garrison-Desany 2022(100) | USA/North America | Prospective cohort study | Hospital/University | Maternal Self-Report, NOWS as Indicator | After birth | Non-medical, MOUD | Illegal, Legal | No | Toddlers and Early Childhood  (18months-6 years old)  Middle Childhood (6-12 years old) | Adjusted for: maternal race and ethnicity, age, education, marital status, and pre-pregnancy BMI, annual household income, parity and child biological sex and prenatal exposure to other substances. | No | Fair | 3138 (60) | Cox proportional hazards model, Bayesian Kernel Machine Regression (BKMR) to assess the effect of exposure to multiple substances | Trained research staff |
| Grattan 1996(105) | USA/North America | Prospective cohort study | Hospital/University | Clinical Record | Not specified | Non-medical, MOUD, Prescribed | Illegal, Prescribed, Legal | No | Middle childhood  (6-12 years old) | Adjusted for:  biological sex and age of children | No | Good | 76 (35) | Analyses of covariance (ANCOVA); Summary descriptive statistics | Author(s) |
| Haabrekke 2018(73) | Norway /Europe | Retrospective cohort study | Opioid Centre/Drug Program | Maternal Self-Report | 1^st^ Trimester, 2^nd^ Trimester, 3^rd^ Trimester, | Not specified | Illegal, Prescribed, Legal | 1st Trimester, 2nd Trimester, 3rd Trimester | Toddlers and Early Childhood  (18months-6 years old) | None | No | Poor | 66 (20) | Linear regression and ANOVA | Parent, Teacher(s) |
| Hall 2019(89) | USA/North America | Retrospective cohort study | Hospital/University | Biological Sample | Time of Delivery | MOUD, Not specified | Not specified | No | Infants and Toddlers  (<18 months old)  Toddlers and Early Childhood  (18months-6 years old) | None | Yes | Poor | 15.544 (611) | Chi-squared tests for categorical diagnosis variables. | Not Specified |
| Hans 2001(35) | USA/North America | Prospective cohort study | Hospital/University | Maternal Self-Report, Biological Sample | 1^st^ Trimester, 2^nd^ Trimester, 3^rd^ Trimester, | Non-medical, MOUD, Prescribed | Illegal, Legal | No | Infants and Toddlers  (<18 months old)  Toddlers and Early Childhood  (18months-6 years old) | Use of four substances other than opioids, cumulative social-environmental risks, and birth weight | Yes | Good | 78 (33) | Repeated measures analyses, ANOVA to compare means and variances | Trained research staff (blind) |
| Hart 2019(67) | USA/North America | Retrospective cohort study | Hospital/University | Maternal Self-Report, Biological Sample | After Birth | MOUD | Illegal, Prescribed, Legal | No | Infants and Toddlers  (<18 months old) | None | No | Poor | 18 (6) | Chi-squared or Fisher’s exact test, whereas two-sample t-tests. Wilcoxon rank-sum test | Not specified |
| Hunt 2008(60) | Australia/ Oceania | Prospective cohort study | Hospital/University | Biological Sample | Not Specified | MOUD | Not specified | No | Toddlers and Early Childhood  (18months-6 years old) | Matched on: maternal age, maternal height, ethnicity, and previous obstetric history | Yes | Poor | 236 (133) | Not specified (T-tests) | Trained pediatricians/physicians, pediatric neurologists, psychologists, developmental psychologists or psychometrists |
| Jaekel 2021(90) | New Zealand/ Oceania | Prospective cohort study | Opioid Centre/Drug Program | Maternal self-report, biological sample, clinical record | 3^rd^ Trimester, at birth | MOUD, not specified | Illegal, Prescribed, Legal | No | Toddlers and Early Childhood  (18months-6 years old)  Middle childhood (6-12 years old) | Adjusted for: child age (squared) at assessment, biological sex,  prenatal biological and social risk, the postnatal rearing  environment, and primary caregiver change | No | Good | 210 (100) | Summary descriptive statistics; Linear mixed effects model | Parent |
| Johnson 1982(37) | USA/North America | Prospective cohort study | Opioid Centre/Drug Program | Biological Sample | Time of Delivery | Non-medical, MOUD | Illegal, Prescribed, Legal | No | Infants and Toddlers  (<18 months old) | Matched on: Child biological sex, birth weight and gestational age | Yes | Fair | 90 (59) | ANOVA, not specified (t-tests) | Trained research staff (blind), Trained examiner(s)(blind) |
| Johnson 1985(36) | USA/North America | Prospective cohort study | Opioid Centre/Drug Program | Biological Sample | Time of Delivery | Non-medical, MOUD | Legal, Illegal, Prescribed | No | Infants and Toddlers  (<18 months old) | Matched on: Child biological sex, birth weight and gestational age | Yes | Fair | 93 (61) | T-tests and Fisher’s Exact Probability Test. | Trained research staff (blind) |
| Kaltenbach 1987(38) | USA/North America | Prospective cohort study | Hospital/University | Clinical Record | Not specified | MOUD | Not specified | No | Infants and Toddlers  (<18 months old) | Matched on: Maternal age, race, and SES | Yes | Poor | 268 (141) | T-test | Trained pediatricians/physicians, pediatric neurologists, psychologists, developmental psychologists or psychometrists |
| Kaltenbach 1989(39) | USA/North America | Prospective cohort study | Other/Not Specified | Not specified | After Birth | MOUD, Not specified | Not specified | No | Infants and Toddlers  (<18 months old)  Toddlers and Early Childhood  (18months-6 years old) | Matched on: Maternal age, race, and SES | No | Poor | 44 (27) | T-test | Not specified |
| Kim 2021(74) | New Zealand/ Oceania | Prospective cohort study | Opioid Centre/Drug Program | Maternal self-report, biological sample, clinical record | 3^rd^ trimester, at birth | MOUD, not specified | Legal, Illegal, Prescribed | No | Toddlers and Early Childhood  (18months-6 years old) | Adjusted for: biological sex, maternal education, non-opioid co-exposures; maternal self-reported depression symptoms; and the quality of maternal nutrition during pregnancy | No | Good | 210 (100) | T-test or chi-squared test of independence | Trained research staff (blind) |
| Konijnenberg 2013(75) | Norway /Europe | Prospective cohort study | Opioid Centre/Drug Program | Clinical Record | 1^st^ Trimester, 2^nd^ Trimester, 3^rd^ Trimester, | Non-medical, MOUD | Legal, Illegal, Prescribed | No | Toddlers and Early Childhood  (18months-6 years old) | Adjusted for: Maternal Education  Matched on: the child’s age, the maternal age and gender | Yes | Poor | 30 (15) | T-tests and ANOVA | Trained research staff (blind) |
| Konijnenberg 2015(76) | Norway /Europe | Prospective cohort study | Opioid Centre/Drug Program | Clinical Record | 1^st^ Trimester, 2^nd^ Trimester, 3^rd^ Trimester, | Non-medical, MOUD | Legal, Illegal, Prescribed | No | Toddlers and Early Childhood  (18months-6 years old) | Adjusted for: birth weight, gestational age, and maternal education and employment. | Yes | Fair | 66 (35) | ANOVA for continuous variables and Pearson’s chi-squared tests for categorical variables. Linear regression | Parent, Teacher(s), Examiner(s) |
| Konijnenberg 2016(53) | Norway /Europe | Prospective cohort study | Opioid Centre/Drug Program | Clinical Record | 1^st^ Trimester, 2^nd^ Trimester, 3^rd^ Trimester, | Non-medical, MOUD | Legal, Illegal, Prescribed | No | Infants and Toddlers  (<18 months old)  Toddlers and Early Childhood  (18months-6 years old) | Adjusted for: for birth weight. | Yes | Fair | 67 (35) | Eta-squared as sum of squares between-groups/total sum of squares interpreted as 0.01 = small effect size, 0.06 = medium effect size, and 0.14 = large effect | Parent, Examiner(s) |
| Konijnenberg 2015(91) | Norway /Europe | Prospective cohort study | Opioid Centre/Drug Program | Clinical Record | 1^st^ Trimester, 2^nd^ Trimester, 3^rd^ Trimester, | Non-medical, MOUD | Legal, Illegal, Prescribed | No | Toddlers and Early Childhood  (18months-6 years old) | Adjusted for: children biological sex and age, birth weight, maternal employment, and education | Yes | Fair | 56 (31) | Eta-squared as sum of squares between-groups/total sum of squares interpreted as 0.01 = small effect size, 0.06 = medium effect size, and 0.14 = large effect | Parent, Examiner(s) |
| Carolien 2021( Konijnenberg 2021)(99) | Norway /Europe | Prospective cohort study | Opioid Centre/Drug Program | Maternal self-report, biological sample | 3^rd^ Trimester | MOUD, prescribed | Illegal, legal, prescribed | No | Middle childhood (6-12 years old) | None | Yes | Poor | 41 (20) | ANOVA or chi-squared test | Not specified |
| Lee 2019(96) | New Zealand/  Oceania | Prospective cohort study | Hospital/University | Clinical Record | 3^rd^ Trimester, Time of Delivery | MOUD, Not specified | Legal, Illegal, Prescribed | No | Middle childhood (6-12 years old) | Matched on: Expected date of birth  Adjusted for: Maternal education, any maternal use of cigarettes, benzodiazepines, alcohol, or cannabis during pregnancy, and maternal depression score at term | No | Good | 184 (85) | T-tests and chi squared tests, multivariable logistic regression analysis. | Trained research staff, graduate students (blind), graduate students (blind) |
| Lee 2020(77) | New Zealand/  Oceania | Prospective cohort study | Hospital/University | Clinical Record | 3^rd^ Trimester, Time of Delivery | MOUD, Not specified | Legal, Illegal, Prescribed | No | Toddlers and Early Childhood  (18months-6 years old) | Matched on: Expected date of birth  Adjusted for: maternal social risk, depression during pregnancy, infant clinical factors, and the quality of the postnatal caregiving environment | No | Good | 192 (89) | T-tests and chi-squared tests. Multivariable Poisson regression adjusted for covariates | Trained research staff (blind) |
| Lesser Katz 1982(54) | USA/North America | Retrospective cohort study | Hospital/University | Not Specified | After birth | Non-medical, MOUD | Prescribed, Not specified | No | Infants and Toddlers  (<18 months old) | None | Yes | Poor | 24 (10) | T-tests | Not specified |
| Lester 2002(55) | USA/North America | Prospective cohort study | Hospital/University | Maternal Self-Report, Biological Sample | 1^st^ Trimester, 2^nd^ Trimester, 3^rd^ Trimester, Time of Delivery | Not specified | Illegal, Legal | No | Infants and Toddlers  (<18 months old) | Matched on: Race, biological sex, and gestational age  Adjusted for: maternal alcohol use, maternal cannabis use, maternal tobacco use, birth weight, social class, and study site | No | Good | 1388 (115) | ANOVA and chi-squared tests. | Trained pediatricians/physicians, pediatric neurologists, psychologists, developmental psychologists or psychometrists, research staff (blind) |
| Levine 2018(92) | New Zealand/Oceania | Prospective cohort study | Opioid Centre/Drug Program | Biological Sample, Maternal Self-Report, Clinical Record | 2^nd^ Trimester, 3^rd^ Trimester, Time of Delivery | MOUD, Not specified | Illegal, Prescribed, Legal | No | Toddlers and Early Childhood  (18months-6 years old) | None | Yes | Poor | 156 (68) | T-tests, chi-squared tests, ANOVA Linear Regression | Trained research staff |
| Levine 2021(78) | New Zealand/  Oceania | Prospective cohort study | Opioid Centre/Drug Program | Maternal self-report, biological sample, clinical record | 3^rd^ trimester, at birth | MOUD, not specified | Legal, Illegal, Prescribed | No | Toddlers and Early Childhood  (18months-6 years old) | Adjusted for: Maternal education and other legal and illegal drug use during pregnancy | No | Good | 200 (92) | T-tests, chi-squared tests, multiple linear regression | Trained research staff |
| Lifschitz 1985(124) | USA/North America | Prospective cohort study | Hospital/University | Maternal Self-Report, clinical record | 1^st^ Trimester, 2^nd^ Trimester, 3^rd^ Trimester, Time of Delivery | Non-medical, MOUD | Legal, Illegal, Not specified | No |  | Matched on: the extent of prenatal care, maternal age, race, and SES  Adjusted for prenatal care score, HOME score, Prenatal risk score | No | Good | 92 (51) | T-tests, ANOVA, multiple regression analysis | Trained pediatricians/physicians, pediatric neurologists, psychologists, developmental psychologists or psychometrists (blind) |
| Lowe 2017(56) | USA/North America | Prospective cohort study | Hospital/University | Biological Sample, Maternal Self-Report, Clinical Record | 1^st^ Trimester, 2^nd^ Trimester, 3^rd^ Trimester, | MOUD, Not specified | Illegal, Legal | No | Infants and Toddlers  (<18 months old) | Adjusted for: Study group, Still-face paradigm episode, infant age at assessment,  infant biological sex, household income, maternal depression score, maternal education, and marital status | No | Good | 144 (113) | ANOVA, Fisher exact test, Welch's modified F-test, MANOVA, linear mixed effect models | Trained pediatricians/physicians, pediatric neurologists, psychologists, developmental psychologists or psychometrists; research staff (blinded) |
| Marcus 1982(68) | USA/North America | Prospective cohort study | Other/Not Specified | Not specified | Not specified | MOUD, Not specified | Not specified | No | Infants and Toddlers  (<18 months old) | None | No | Poor | 38 (15) | Summary descriptive statistics | Trained examiner(s)(blind) |
| McGlone 2015(40) | United Kingdom/  Europe | Prospective cohort study | Hospital/University | Maternal Self-Report, Biological Sample | Time of Delivery | Non-medical, MOUD | Legal, Illegal, Prescribed | No | Infants and Toddlers  (<18 months old) | Matched on: gestation, birth weight and postcode  at delivery | Yes | Good | 107 (81) | Mann–Whitney; Kruskal–Wallis; Multivariable Linear regression models | Author(s) |
| Melinder 2013(79) | Norway/  Europe | Retrospective cohort study | Opioid Centre/Drug Program | Clinical record | 1^st^ Trimester, 2^nd^ Trimester, 3^rd^ Trimester | MOUD | Legal | No | Toddlers and Early Childhood  (18months-6 years old) | Adjusted for: Motor skills, birth weight and maternal education and employment  Matched on: gender and age. | No | Poor | 49 (26) | Fisher’s exact test; ANOVA | Parent, Trained Research Staff |
| Messinger 2004(41) | USA/North America | Prospective cohort study | Hospital/University | Maternal Self-Report, Biological Sample | 1^st^ Trimester, 2^nd^ Trimester, 3^rd^ Trimester, Time of Delivery | Not specified | Legal, Illegal | No | Infants and Toddlers  (<18 months old)  Toddlers and Early Childhood  (18months-6 years old) | Matched on: Ethnicity, biological sex, gestational age  Adjusted for: birth weight, SES, maternal education, vocabulary size, race, and  psychopathology, as well as prenatal exposure to alcohol, cigarettes, and cannabis | No | Good | 1227 (98) | Univariate ANOVA; Hierarchical linear modeling; Chi-squared analyses | Trained examiner(s)(blind) |
| Moe 2002(69) | Norway/  Europe | Prospective cohort study | Opioid Centre/Drug Program | Maternal Self-Report, clinical record | 2^nd^ Trimester, 3^rd^ Trimester | Non-medical | Illegal, Legal, Prescribed | No | Infants and Toddlers  (<18 months old)  Toddlers and Early Childhood  (18months-6 years old) | Adjusted for: gestational age, parental SES, mental development index at age 1 | No | Poor | 116 (64) | T-tests and two-way ANCOVA; Mann-Whitney U Test; Chi-squared tests; Multivariable linear regression | Trained examiner(s) |
| Nair 2008(80) | USA/North America | Retrospective cohort study | Registry linkage study | Biological sample, Maternal Self-Report | After Birth | Non-medical | Legal, Illegal | No | Toddlers and Early Childhood  (18months-6 years old)  Middle childhood (6-12 years old) | Matched on: SES, age of first pregnancy, and race  Adjusted for biological sex, prenatal tobacco exposure, number of caregiver placement changes, and 3 caregiver variables  assessed at age 7, including depressive symptoms, employment  status, and public assistance status | Yes | Good | 173 (111) | MANOVA; ANOVA. | Trained research staff (blind) |
| Nygaard 2016(101) | Norway/Europe | Retrospective cohort study | Opioid Centre/Drug Program | Maternal Self-Report, clinical record | 2^nd^ Trimester, 3^rd^ Trimester, After Birth | Non-medical, Not specified | Prescribed, Legal, Illegal | No | Middle childhood (6-12 years old) | Adjusted for: Child biological sex and relative age at the time of assessment, SES, gestational age, and birth weight | No | Poor | 130 (72) | T-test; Mann-Whitney U test; Linear mixed effects models | Author(s), clinical psychology graduates |
| Nygaard 2015(42) | Norway/Europe | Prospective cohort study | Opioid Centre/Drug Program | Maternal Self-Report, Clinical Record | 2^nd^ Trimester, 3^rd^ Trimester, After Birth | Non-medical, Not specified | Prescribed, Legal, Not specified | No | Infants and Toddlers  (<18 months old)  Toddlers and Early Childhood  (18months-6 years old)  Middle childhood (6-12 years old) | Adjusted for: Earlier cognitive ability scores, gestational age, age at assessment, biological sex, birthweight, SES | No | Good | 130 (72) | Mixed effects models; Multivariable regression | Author(s), clinical psychology graduates |
| Oei 2017(97) | Australia/Oceania | Retrospective cohort study | Registry linkage study | NOWS as indicator | After Birth | Not specified | Not specified | No | Middle childhood (6-12 years old) | Matched on: gestation, socioeconomic status, and biological sex | Yes | Good | 6.564 (2.234) | Chi-squared and Fisher exact tests ANOVA; Mann–Whitney U test; Binary multivariable logistic regression | Medical diagnosis from dataset |
| Ornoy 1996(43) | Israel/Asia | Retrospective cohort study | Opioid Centre/Drug Program | Clinical Record | After Birth | Non-medical, MOUD | Not specified | No | Infants and Toddlers  (<18 months old)  Toddlers and Early Childhood  (18months-6 years old) | Matched on: children age and SES | Yes | Fair | 213 (83) | T-test, Chi-squared test; ANOVA | Author(s), trained pediatricians/physicians, pediatric neurologists, psychologists, developmental psychologists or psychometrists (partially blind), |
| Ornoy 2001(64) | Israel/Asia | Retrospective cohort study | Opioid Centre/Drug Program | Clinical Record | After Birth | Non-medical | Not specified | No | Toddlers and Early Childhood  (18months-6 years old)  Middle childhood (6-12 years old) | Matched on: children age and SES | No | Good | 160 (65) | MANOVA; | Trained examiner(s) (partially blind) |
| Ornoy 2010(66) | Israel/Asia | Retrospective cohort study | Opioid Centre/Drug Program | Maternal Self-Report, clinical record | After Birth | Non-medical, MOUD | Not specified | No | Adolescence  (>12 years old) | Matched on: children age and SES | Yes | Fair | 191 (55) | MANOVA; ANOVA; T-test | Parent, Trained pediatricians/physicians, pediatric neurologists, psychologists, developmental psychologists or psychometrists (blind) |
| Ornoy 2016(65) | Israel/Asia | Retrospective cohort study | Opioid Centre/Drug Program | Maternal Self-report, Clinical record | After Birth | Non-medical, MOUD | Legal, Not specified | No | Toddlers and Early Childhood  (18months-6 years old)  Middle childhood (6-12 years old) | None | Yes | Poor | 158 (64) | T-test or Wilcoxon signed test; Chi-squared test | Parent, Not specified |
| Pulsifer 2008(81) | USA/North America | Retrospective cohort study | Hospital/University | Maternal Self-Report, Biological Sample | After Birth | Not specified | Illegal, Legal | No | Toddlers and Early Childhood  (18months-6 years old) | Matched on: SES, maternal age, and gestational age. | Yes | Fair | 251 (47) | Chi-squared tests; ANOVA; T- test | Trained examiner(s)(blind) |
| Robbins 2021(82) | USA/North America | Retrospective Cohort Study | Registry linkage study | Prescribed | After birth | Non-medical, Legal, Prescribed | Prescribed | During delivery | Toddlers and Early Childhood  (18months-6 years old) | Adjusted for gestational age at delivery, mode of delivery, maternal education, exposure to magnesium, and exposure to alcohol, cocaine, marijuana, and tobacco | No | Good | 1404 (535) | Chi-square and Fisher exact tests; T-tests or Wilcoxon rank-sum tests; multivariable logistic regression models | Trained pediatricians/physicians, pediatric neurologists, psychologists, developmental psychologists or psychometrists |
| Rosen 1985(70) | USA/North America | Prospective cohort study | Other/Not Specified | Clinical Record, Biological Sample | 1^st^ Trimester, 2^nd^ Trimester, 3^rd^ Trimester, Time of Delivery, After Birth | MOUD, Not specified | Illegal, Prescribed, Legal | No | Toddlers and Early Childhood  (18months-6 years old) | Matched on: Maternal race, SES, biological sex, birth weight, and gestational age | Yes | Poor | 93 (61) | Not Specified | Not specified |
| Rubenstein 2019(83) | USA/North America | Case-Control Study | Hospital/University | Clinical record | After Birth | Non-medical, MOUD | Prescribed, Legal | Before Pregnancy, 1st Trimester, 2nd Trimester, 3rd Trimester, After Birth | Toddlers and Early Childhood  (18months-6 years old) | Adjusted for: Study period of each cohort, maternal race/ethnicity, education, tobacco smoking during pregnancy, and psychiatric conditions  pre-childbirth | Yes | Poor | 1703 (126) | Multivariable Logistic regression | Parent, Examiner(s) |
| Salo 2009(84) | Finland/Europe | Retrospective cohort study | Hospital/University | Maternal Self-Report, Biological Sample | After Birth | MOUD | Illegal, Prescribed, Legal | No | Toddlers and Early Childhood  (18months-6 years old) | None | No | Poor | 34 (21) | One-way ANOVA | Trained pediatricians/physicians, pediatric neurologists, psychologists, developmental psychologists or psychometrists(blind), examiner(s) (blind) |
| Salo 2010(44) | Finland/Europe | Retrospective cohort study | Hospital/University | Maternal Self-Report, Biological Sample | After Birth | MOUD, Not specified | Illegal, Prescribed, Legal | Before Pregnancy, 1st Trimester, 2nd Trimester, 3rd Trimester | Infants and Toddlers  (<18 months old) | Adjusted for: Infant age at testing birth weight and length, gestational age, maternal age and education | No | Poor | 72 (15) | T-tests; chi-squared test; one-way ANOVAs | Trained pediatricians/physicians, pediatric neurologists, psychologists, developmental psychologists or psychometrists |
| Sandtorv 2018(102) | Norway/Europe | Retrospective cohort study | Hospital/University | NOWS as indicator, Biological Sample, Clinical Record | After Birth | Not specified | Not specified | No | Middle childhood (6-12 years old) | Adjusted for: Child age, biological sex, IQ, and child placement outside of their biological home  before or after the age of 1 year.  Matched on: children age and biological sex | No | Poor | 228 (57) | T-test; Multivariable regression analysis | Parent, Not specified |
| Sarfi 2013(93) | Norway/Europe | Prospective cohort study | Opioid Centre/Drug Program | Maternal Self-Report, Biological Sample | 3^rd^ Trimester | Non-medical, MOUD | Illegal, Legal | Before Pregnancy, 1st Trimester, 2nd Trimester, 3rd Trimester | Toddlers and Early Childhood  (18months-6 years old) | Adjusted for: Maternal education, child birthweight, child gender, child mother interaction measure, parenting stress index, maternal depression | No | Fair | 68 (33) | T-test; Multivariable régression analyses | Parent, Trained examiner(s)(blind), Not specified |
| Sarfi 2021(104) | Norway/Europe | Prospective cohort study | Opioid Centre/Drug Program | Maternal Self-Report, Biological Sample | 3^rd^ trimester | MOUD, prescribed | Illegal, Prescribed, Legal | No | Middle childhood (6-12 years old) | None | No | Poor | 218 (78) | ANOVA | Parent, Teacher(s) |
| SerinoMa 2018(45) | USA/North America | Prospective cohort study | Hospital/University | Maternal Self-Report, Biological Sample | 1^st^ Trimester, 2^nd^ Trimester, 3^rd^ Trimester | Non-medical, MOUD | Illegal, Legal | Before Pregnancy,  1^st^ Trimester, 2^nd^ Trimester, 3^rd^ Trimester , After Birth | Infants and Toddlers  (<18 months old)  Toddlers and Early Childhood  (18months-6 years old) | Adjusted for socioeconomic status | Yes | Fair | 73 (24) | ANOVA; maternal age. Multivariable Linear and logistic regression; chi-squared test | Author(s) (blind) |
| Skovlund 2017(125) | Norway/Europe | Prospective cohort study | Registry linkage study | Maternal Self-report | 1^st^ Trimester, 2^nd^ Trimester, 3^rd^ Trimester | MOUD, Prescribed | Illegal, Prescribed, Legal | Before Pregnancy, 1st Trimester, 2nd Trimester, 3rd Trimester, After Birth | Toddlers and Early Childhood  (18months-6 years old) | Adjusted for: maternal employment, paternal education, maternal BMI, parity, maternal smoking, benzodiazepine, and maternal SSRI use during pregnancy | No | Good | 45.646 (804) | Ordinal (proportional odds), multinomial and binary logistic regression models | Parent |
| Skovlund 2020(85) | Norway/Europe | Prospective cohort study | Registry linkage study | Maternal Self-report | 1^st^ Trimester, 2^nd^ Trimester, 3^rd^ Trimester, After Birth | Not specified, Prescribed | Illegal, Prescribed, Legal | Before Pregnancy, 1st Trimester, 2nd Trimester, 3rd Trimester, After Birth | Toddlers and Early Childhood  (18months-6 years old) | Adjusted for: Paracetamol, any pain, chronic disease, planned pregnancy, smoking, mother's education, father's education, mother's employment, mother's age, father's age, parity, marital status, BMI, alcohol consumption, anxiety and depression (mean HSCL), illegal drugs, benzodiazepine use, triptan use, SSRI use | No | Good | 30.584 (584) | Ordinal (proportional odds), multinomial and binary logistic regression models | Parent |
| Skumlien 2020(46) | Denmark/Europe | Retrospective cohort study | Opioid Centre/Drug Program | Maternal Self-Report, Biological Sample | Not specified | Non-medical, MOUD, Prescribed | Illegal, Prescribed, Legal | No | Infants and Toddlers  (<18 months old) | Adjusted for: biological sex, Prenatal Cannabis Exposure, Age and Maternal Education | Yes | Fair | 132 (94) | Linear mixed models  . | Not Specified |
| Slinning 2004(63) | Norway/Europe | Prospective cohort study | Opioid Centre/Drug Program | Maternal Self-Report, Clinical Record, Biological Sample | 1^st^ Trimester, 2^nd^ Trimester, 3^rd^ Trimester, After Birth | Non-medical | Illegal, Legal, Prescribed | No | Infants and Toddlers  (<18 months old)  Toddlers and Early Childhood  (18months-6 years old) | Matched on: Children’s age | No | Fair | 92 (42) | T-test and two-way ANOVA; ANCOVA | Trained pediatricians/physicians, pediatric neurologists, psychologists, developmental psychologists or psychometrists, Parent, Teacher |
| Strauss 1975(57) | USA/North America | Prospective cohort study | Hospital/University | Clinical Record | 1^st^ Trimester, 2^nd^ Trimester, 3^rd^ Trimester | Non-medical, MOUD | Not specified | No | Infants and Toddlers  (<18 months old) | Matched on: neighborhood, public medical assistance programs | Yes | Poor | 66 (22) | mixed-design ANOVA; Chi-squared test statistics | Trained examiner(s) |
| Strauss 1976(48) | USA/North America | Prospective cohort study | Opioid Centre/Drug Program | Clinical Record | 1^st^ Trimester, 2^nd^ Trimester, 3^rd^ Trimester | Non-medical, MOUD | Not specified | No | Infants and Toddlers  (<18 months old) | Matched on: birth weight, gestational age, one- and five- minute Apgar scores, potency of obstetric analgesia and anesthesia, and number of prenatal clinic visits | Yes | Poor | 113 (60) | ANOVA | Trained examiner(s) |
| Strauss 1979(47) | USA/North America | Prospective cohort study | Hospital/University | Clinical Record | After Birth | MOUD | Not specified | No | Infants and Toddlers  (<18 months old)  Toddlers and Early Childhood  (18months-6 years old) | Matched on: birth weight, gestational age, one- and five- minute Apgar scores, potency of obstetric analgesia and anesthesia, and number of prenatal clinic visits | Yes | Poor | 63 (33) | F-statistics and T-tests | Trained pediatricians/physicians, pediatric neurologists, psychologists, developmental psychologists or psychometrists |
| Trønnes 2021(103) | Norway/Europe | Prospective cohort study | Registry linkage study | Maternal Self-report, Clinical Record | 1^st^ trimester, 3^rd^ trimester | Prescribed | Illegal, Legal, Prescribed | 1^st^ Trimester, 2^nd^ Trimester, 3^rd^ Trimester, | Middle childhood (6-12 years old) | Adjusted for:  maternal age, marital status, maternal education, maternal income, parity, pre-pregnancy BMI, consumption of folic acid supplement, tobacco smoking and alcohol use, illegal drug use, maternal chronic conditions in early pregnancy, symptoms of anxiety and depression frequency of pain episodes and familial history of ADHD | Yes | Good | 73.480 (1726) | Inverse probability of treatment weights (IPTW); Cox Proportional Hazard Models | Parent |
| vanBaar 1989(49) | The Netherlands/Europe | Prospective cohort study | Hospital/University | Maternal Self-Report, Biological Sample | Not specified | Non-medical, MOUD | Illegal, Legal, Not specified | No | Infants and Toddlers  (<18 months old) | None | No | Poor | 72 (35) | Kruskal-Wallis analysis of variance; Fisher's exact test | Trained Examiner(s) |
| vanBaar 1989(59) | The Netherlands/Europe | Prospective cohort study | Hospital/University | Maternal Self-Report, Biological Sample | Not specified | Non-medical, MOUD | Illegal, Legal, Not specified | No | Infants and Toddlers  (<18 months old) | None | No | Poor | 72 (35) | Kruskal-Wallis analysis of variance; Fisher's exact test | Parent, Trained Examiner(s) |
| vanBaar 1990(50) | The Netherlands/Europe | Prospective cohort study | Hospital/University | Maternal Self-Report, Biological Sample | After Birth | Non-medical, MOUD | Illegal, Legal, Not specified | No | Infants and Toddlers  (<18 months old)  Toddlers and Early Childhood  (18months-6 years old) | None | Yes | Poor | 72 (35) | T-test; ANOVA for repeated measurements; Chi-squared test | Parent, Trained Examiner(s) |
| vanBaar 1994(61) | The Netherlands/Europe | Prospective cohort study | Hospital/University | Maternal Self-report, Biological Sample, Clinical Record | 1^st^ Trimester, 2^nd^ Trimester, 3^rd^ Trimester, After Birth | Non-medical, MOUD | Illegal, Prescribed, Legal | No | Toddlers and Early Childhood  (18months-6 years old) | None | Yes | Poor | 58 (23) | T-test; ANOVA for repeated measurements; Chi-squared test; ANCOVA; Mann-Whitney u test | Parent, Trained Examiner(s) |
| Walhovd 2010(98) | Norway/Europe | Prospective cohort study | Hospital/University | Maternal Self-Report, clinical record | After Birth | Non-medical, Not Specified | Illegal, Prescribed, Legal | 1^st^ trimester, 2^nd^ trimester, 3^rd^ Trimester, | Middle childhood  (6-12 years old) | Adjusted for children age and biological sex | Yes | Poor | 28 (14) | One-way ANOVA | Not specified |
| Walhovd 2015(86) | Norway/Europe | Prospective cohort study | Opioid Centre/Drug Program | Maternal Self-Report, Biological Sample | Not Specified | Non-medical, Not Specified | Illegal, Prescribed, Legal | 2^nd^ trimester, 3^rd^ trimester | Toddlers and Early Childhood  (18months-6 years old) | Adjusted for children age and biological sex | Yes | Poor | 24 (12) | One-way ANOVA | Not specified |
| Wen 2021(87) | USA/North America | Retrospective cohort study | Registry linkage study | Clinical Record | After delivery | Legal, Prescribed | Legal, Prescribed | Yes | Toddlers and Early Childhood  (18months-6 years old) | Adjusted for: Maternal age at birth, birth year, and location, Obstetric characteristics, number of gestations, legal and illegal other substance abuse, obesity, Maternal comorbid conditions and index, other medication use, number of in-patient hospitalizations, number of days of hospitalization, number of outpatient visits | No | Good | 24.910 (1899) | Cox proportional-hazard models; propensity score methods for adjustment | Medical diagnosis from dataset |
| Wilson 1979(88) | USA/North America | Retrospective cohort study | Hospital/University | Clinical Record | After Birth | Non-medical, MOUD | Prescribed, Not specified | No | Toddlers and Early Childhood  (18months-6 years old) | Matched on: Maternal age, race, and SES and child biological sex | No | Fair | 77 (22) | Multivariable linear regression | Author(s), parent |
| Wilson 1981(51) | USA/North America | Prospective cohort study | Hospital/University | Maternal Self-report, Biological Sample, Clinical Record | 1^st^ Trimester, 2^nd^ Trimester, 3^rd^ Trimester, Time of Delivery | Non-medical, MOUD, Prescribed | Illegal, Prescribed, Legal | No | Infants and Toddlers  (<18 months old) | Matched on: maternal age, race, SES, marital status, and duration of gestation | No | Good | 126 (69) | T-test and chi-squared test | Author(s), Not specified |
| Wouldes 2020(58) | New Zealand /Oceania | Prospective cohort study | Hospital/University | Maternal Self-Report, Biological Sample | 1^st^ Trimester, 2^nd^ Trimester, 3^rd^ Trimester | MOUD, Not specified | Illegal, Prescribed, Legal | No | Infants and Toddlers  (<18 months old) | Adjusted for: corrected age of test assessment, socioeconomic status, maternal prescription drug and psychoactive legal and illegal drug use during pregnancy including medications prescribed (SSRIs, benzodiazepines & Ritalin), tobacco smoking, alcohol and cannabis use, average times benzodiazepines, other opiates or stimulants were used per week; birthweight, sex and gestational age in completed weeks; number of terminations, gravida and documented or self-reported history of mental illness | No | Good | 189 (86) | T-tests, Kruskal-Wallis, and Chi-square statistics clinical differences at birth. Generalized linear models GLIM  Latent profile analysis (LPA) to classify children into homogenous groups | Trained research staff |

**^a^** Non-medical opioids were: Heroin, fentanyl, opium or any other opioid that were not used for medical purposes; Medication for Opioid Use Disorder (MOUD) were: Methadone Maintenance Therapy; Buprenorphine Therapy, or other MOUD; Prescribed opioids were: Tramadol, Oxycodone, Codeine, Morphine and other opioids prescribed by a health professional for medical use

^b^ Illegal co-exposures were Cocaine, Crack, LSD, Cannabis, Phencyclidine; Prescribed co-exposures were: Amphetamines, SSRI anti-depressants, Barbiturates, and Benzodiazepines and other medications; Legal co-exposures were: Alcohol and Tobacco

^c^ Trained research staff include” Research Assistants, Study coordinators, Study clinical staff.

^d^ Examiners include: Coders, observers, testers trained to measure neurodevelopment

### Supplemental Table 7: Studies Investigating Prenatal Exposure to Opioids and Cognitive Development

| **Type of Test** | **Author’s name^10^** | **Quality Assessment** | **Age of Testing** | **Findings**  **Mean (SD)** |
| --- | --- | --- | --- | --- |
| Ages and Stages Questionnaire 5 (ASQ5) | Skovlund 2020 | Good | 5 years | Exposed children’s (n=584) adjusted odds ratio of lower (mean score ≤ 7.5) vs best possible (mean score 10) score was 0.82 (0.57, 1.08) compared to unexposed children (n=30,000) |
| Ages and Stages Questionnaire 3 years | Skovlund 2017 | Good | 3 years | Exposed children’s (n=804) adjusted odds ratio of lower communication skills score was 1.10 (0.96, 1.27) compared to unexposed children.  Exposed children’s (n=804) adjusted odds ratio of lower language competences score was 1.04 (0.89, 1.22) compared to unexposed children. |
| Bender gestalt test | Davis 1988 | Good | 6-15 years | Exposed children in utero (n=28) mean score was 6.95 (3.42) vs. unexposed children (n=28) had mean score of 2.54 (2) |
|  | Konijenberg 2013 | Poor | 4.5 years | The mean score in “Perception” domain among preschool children exposed to opioids in utero (n=15) was 4.53(1.41) vs. 5.33(1.45) among unexposed preschool children(n=15). |
|  | Ornoy 2001 | Good | 5-12 years | The average years of delay in achievements on among exposed children in utero(n=65) was 0.87(1.45)^8^, compared to unexposed children from low SES families(n=32) with 1.27 (1.33) and unexposed children (n=30) from normal SES families with 0.05(1.43) years of delay. |
| BRIEF-P  (Behavior Rating Inventory of Executive Function - Preschool version) | Konijnenberg 2015 | Poor | 4 ½ years | Exposed children’s (n=35) mean “working memory” domain score was 57.46 (11.37) vs. unexposed children (n=31) had mean “working memory” domain score of 49.13 (9.98). |
| BSID-III  cognitive scale | Bakhireva 2019 | Poor | 5-8 months | Exposed infants’ (n=42) mean score was 101.29 (8.32) vs. unexposed infants (n=36) had mean score of 100.8(9.4) |
|  | Beckwith 2015 | Poor | 1-42 months | Exposed children(n=28) mean score was 90.18 (11.43) vs. unexposed children (n=1700) had mean score of 100 (15) |
|  | Salo  2009 | Poor | 3 years | Exposed toddler’s(n=28) mean score was 8.9 (0.94) vs. unexposed toddlers (n=13) had mean score of 10.54 (1.26) |
|  | Salo  2010 | Poor | 8-12 months | Exposed infants’ (n=15) mean score was 92.33 (10.73) vs. unexposed infants(n=57) had mean score of 105.11 (7.61) |
|  | SerinoMa 2018 | Fair | 12 months | Exposed infants’ (n=10) mean score was 95.4 (3.9) vs. unexposed infants(n=27) had mean score of 102.6 (2.9) |
|  |  |  | 18-24 months | Exposed infants’ (n=11) mean score was 83.3 (16.4) vs. unexposed infants(n=37) had mean score of 97.6 (10.9) |
|  | Skumlien 2020 | Fair | 12,5 (8.02) months | Boys in the opioid group (n=45) had lower cognitive scores compared to boys in the nonexposed group (n=21) (mean difference −5.82, 95% CI [−9.92, −1.47])  Opioid exposed girls (n=49) scored similar to unexposed girls (n=17) (mean difference −0.03 [−4.48, 4.41] |
| BSID-III  language scale | Bakhireva 2019 | Poor | 5-8 months | Exposed infants’ (n=42) mean BSID score was 101.13 (6.94) vs. unexposed infants had mean score of 98.6(21.4) |
|  | Beckwith 2015 | Poor | 1-42 months | Exposed infants’ (n=28) mean score was 82.12 (12.53) vs. unexposed infants(n=1700) had mean score of 100 (15) |
|  | Salo  2009 | Poor | 3 years | Exposed infants’ (n=28) mean score was 8.9 (0.94) vs. unexposed infants (n=13) had mean score of 23.69 (2.13) |
|  | SerinoMa 2018 | Fair | 12 months | Exposed infants’ (n=10) mean score was 82.9 (5.8) vs. unexposed infants (n=27) had mean score of 86.9 (2.3) |
|  |  |  | 18-24 months | Exposed infants’ (n=11) mean score was 78.3 (16.1) vs. unexposed infants (n=37) had mean score of 85.8 (13.6) |
|  | Skumlien 2020 | Fair | 11.4 (6.94) months | Boys in the opioid group (n=45) had lower language scores compared to boys in the nonexposed group (n=21) (mean difference -3.98, 95% CI [−7.96, 0.00])  Opioid exposed girls (n=49) scored higher compared to unexposed girls (n=17) (mean difference −2.65 [−1.66, 6.96] |
| BSID-II Mental Development Index (MDI) | Bauer 2020 | Good | 1, 2, and 3 years | Exposed infants (n=45) had significantly lower scores (p=0.03) for continuous variables, p<0.01 for categorical variables) than unexposed infants |
|  | Bernstein 1984 | Poor | 4 months | Exposed infants’ (n=17) mean score was 109.9 (12.8) vs. unexposed infants(n=23) had mean score of 115 (14.7) |
|  | Chasnoff  1986 | Fair | 3 months | Exposed infants’ (n=36) mean score was 104.2 (11.1) vs. unexposed infants(n=34) had mean score of 99.2 (14.7) |
|  |  |  | 6 months | Exposed infants’ (n=26) mean score was 103.6 (13.5) vs. unexposed infants(n=29) had mean score of 111 (12.3) |
|  |  |  | 12 months | Exposed infants’ (n=20) mean score was 99.6 (10.6) vs. unexposed infants(n=27) had mean score of 105.8 (8.1) |
|  |  |  | 24 months | Exposed infants’ (n=16) mean score was 98.7 (16) vs. unexposed infants(n=14) had mean score of 96.2 (15.9) |
|  | Hans 2001 | Good | 4 months | Exposed infants’ (n=33) mean score was 111 (12.3) vs. unexposed infants(n=45) had mean score of 114 (15.1) |
|  |  |  | 8 months | Exposed infants’ (n=33) mean score was 116 (19.5) vs. unexposed infants(n=45) had mean score of 120(20.2) |
|  |  |  | 12 months | Exposed infants’ (n=33) mean score was 107 (14.3) vs. unexposed infants(n=45) had mean score of 109 (13.7) |
|  |  |  | 18 months | Exposed toddlers (n=16) mean score was 95 (16.3) vs. unexposed toddlers (n=45) had mean score of 103 (13.1) |
|  |  |  | 24 months | Exposed toddlers (n=36) mean score was 92 (12.7) vs. unexposed toddlers (n=45) had mean score of 96 (12.3) |
|  | Hunt 2008 | Poor | 19.2 months | Exposed toddlers (n=79) mean score was 88.2 (16.4) vs. unexposed toddlers (n=61) had mean score of 105.2 (23) |
|  | Johnson 1982 | Fair | 6 months | Exposed infants’ (n=39) mean score was 95.8 (16.1) vs. unexposed infants(n=23) had mean score of 100.7 (20.1) |
|  | Johnson 1985 | Fair | 12 months | Exposed infants’ (n=46) mean score was 97.9 (2.68) vs. unexposed infants(n=22) had mean score of 107 (2.81) |
|  | Kaltenbach 1987 | Poor | 12 months | Exposed infants’ (n=105) mean score was 103.53 (NA) vs. unexposed infants(n=63) had mean score of 104.39 (NA) |
|  | Kaltenbach 1989 | Poor | 6 months | Exposed infants’ (n=27) mean score was 107.9 (12.23) vs. unexposed infants(n=17) had mean score of 105.6 (7.31) |
|  |  |  | 12 months | Exposed infants’ (n=27) mean score was 102.5 (11.38) vs. unexposed infants(n=17) had mean score of 106.53 (6.41) |
|  |  |  | 24 months | Exposed toddlers (n=27) mean score was 100.9 (18.04) vs. unexposed toddlers (n=17) had mean score of 103.92 (11.49) |
|  | Levine 2021^1^ | Poor | 24 months | Methadone exposed toddlers (n=92) mean score was 77.48 (18.04) vs. unexposed toddlers (n=108) had mean score of 92.35 (16.30).  Additionally, the proportion of exposed children with cognitive delays (≤ 1 SD comparison group mean MDI score) was significantly higher compared to unexposed children (51.1% vs. 13.9%). Linear Regression models suggested that the children exposed to opioids had lower cognitive development score compared to non- exposed children after adjusting for confounders (b=−6.85; 95%CI: −13.71, 0.01). |
|  | Messinger 2004 | Good | 12 months | Exposed infants’ (n=79) mean score was 88.5(1.2) vs. unexposed infants(n=960) had mean score of 91.6 (0.4) |
|  |  |  | 24 months | Exposed toddlers (n=80) mean score was 82.1 (1.6) vs. unexposed toddlers (n=931) had mean score of 81.7 (0.4) |
|  |  |  | 36 months | Exposed toddlers (n=78) mean score was 83 (1.6) vs. unexposed toddlers (n=918) had mean score of 82.6 (0.4) |
|  | Nygaard2015^2^ | Fair | 12 months | Exposed infants’ (n=66) mean score was 92.21 (14.31) vs. unexposed infants(n=58) had mean score of 98.7 (8.94) |
|  |  |  | 24 months | Exposed toddlers (n=65) mean score was 93.68 (16.8) vs. unexposed toddlers (n=55) had mean score of 101.69 (10.68) |
|  |  |  | 36 months | Exposed toddlers (n=69) mean score was 95.54 (12.52) vs. unexposed toddlers (n=57) had mean score of 102.36 (10.16) |
|  | Ornoy 1996^8^ | Fair | 6-24 months | Exposed infants’ (n=37) mean score was 104 (15.8) vs. unexposed infants from low SES families(n=21) had mean score of 87.3 (9.85) vs. unexposed healthy infants(n=47) had mean score of 112(14.9). |
|  | Rosen 1985^3^ | Poor | 18 months | Exposed toddlers (n=38) mean score was 96 (2.3) vs. unexposed toddlers (n=23) had mean score of 106.4 (3.6) |
|  |  |  | 24 months | Exposed toddlers (n=34) mean score was 90.4 (2.6) vs. unexposed toddlers (n=22) had mean score of 96.9 (3.1) |
|  | Robbins 2021 | Good | 24 months | Higher proportion of exposed children to opioids during delivery exhibited mental development delays compared to unexposed children (33.7% vs. 30.3%). No association was observed between opioid medications use during delivery and any mental delay (OR=0.85, 95%CI: 0.67, 1.09). Results were not differed when stratified by severity of neurodevelopmental delay |
|  | Straus 1976 | Poor | 3 months | Exposed infants’ (n=25) mean score was 112.5 (11.5) vs. unexposed infants(n=26) had mean score of 115.3 (13.5) |
|  |  |  | 6 months | Exposed infants’ (n=25) mean score was 115.7 (16.8) vs. unexposed infants(n=26) had mean score of 114.3 (20.9) |
|  |  |  | 12 months | Exposed infants’ (n=25) mean score was 113.4 (10.2) vs. unexposed infants(n=26) had mean score of 114.8 (11.3) |
|  | Straus 1979 | Poor | 12 months | Exposed infants’ (n=33) mean score was 113.5 (9.4) vs. unexposed infants(n=30) had mean score of 115.1 (11.4) |
|  | VanBaar 1990 ^4^ | Poor | 6 months | Exposed infants’ (n=27) mean score was 106 (13) vs. unexposed infants(n=37) had mean score of 107 (13) |
|  |  |  | 12 months | Exposed infants’ (n=26) mean score was 108 (12) vs. unexposed infants(n=34) had mean score of 114 (17) |
|  |  |  | 18 months | Exposed toddlers (n=22) mean score was 92 (14) vs. unexposed toddlers (n=34) had mean score of 99 (19) |
|  |  |  | 24 months | Exposed toddlers (n=26) mean score was 86 (15) vs. unexposed toddlers (n=34) had mean score of 98 (16) |
|  |  |  | 30 months | Exposed toddlers (n=25) mean score was 87 (18) vs. unexposed toddlers (n=34) had mean score of 101 (20) |
|  | Wilson 1981 | Poor | 9 months | Heroin exposed infants in utero (n=29) mean score was 97.2 (17.6) vs. infants whose mothers were treated with MOUD (n=35) had mean score of 99.3(15.5) and unexposed infants(n=55) had mean score of 105.5 (15.6) |
| Clinical Evaluation of Language Fundamentals – Preschool, Total score | Kim 2021^5^ | Good | 4.5 years | Opioid-exposed children’s (n=89) mean score was 83.6 (14.9), unexposed children (n=103) had a mean score of 100.5 (16.0). In addition, children prenatally exposed to opioids were 3 times more likely to manifest language delay problems (29% vs. 11%). Unexposed children had higher total language development scores after adjusting for covariates compared to the opioid exposed children b=7.87 (0.78, 14.97). |
| Columbia Mental Maturity | Wilson 1979 | Good | 3-6 years | The mean score among children exposed to opioids in utero (n=22) was 96.06 (NA) vs. 99.15 among unexposed children living in drug environment (n=20) vs. 100.51 unexposed children from medically high-risk pregnancies (n=15) vs. 99.48 children living in average SES conditions (n=20). |
| Communication and Symbolic Behavior Scales Developmental Profile | Levine 2021^1^ | Good | 2 years | Completed by parents. The methadone exposed children had significantly lower average language development score compared to unexposed children (49.94 vs. 52.42) Additionally the proportion of exposed children with language delays (total score ≤10^th^ percentile of comparison group) was significantly higher than the unexposed children (24.1% vs. 10.9%). Linear Regression models suggested that the children exposed to opioids did not have a significant lower language development scores compared to unexposed children. (b=−0.90; 95%CI: −2.82,1.02) |
| Constellation/Syndrome of CNS/ANS signs at birth | Bada 2002 | Good | at birth | The adjusted odds having CNS/ANS signs at birth was 2.8 (95%CI: 2.1-3.7) among newborns exposed to opioids in utero compared to unexposed neonates. |
| Developmental Delay | Rubenstein 2019 | Poor | 2-6 years | For children exposed to opioids in the entire peri-pregnancy period adjusted odds of having developmental delays was 1.94 (95%CI: 0.68, 5.52).  For children exposed to opioids in the 1st trimester period adjusted odds of having developmental delays was 1.64 (95%CI: 0.87,3.12).  For children exposed to opioids in the 2nd trimester period adjusted odds of having developmental delays was 0.85 (95%CI: 0.52, 1.41).  For children exposed to opioids in the 3rd trimester period adjusted odds of having developmental delays was 1.06(95%CI: 0.76, 1.49). |
|  | Hall 2019 | Poor | >24 months | Out of children exposed to opioids without developing NAS after birth (n=473), 15.6% were diagnosed with developmental delay. 28.3% of exposed children with NAS after birth (n=138) were diagnosed with developmental delay. In unexposed children (n=14,933), 7.6% were diagnosed with developmental delay. |
|  | Wen 2021 | Good | <18 -36 months | The proportion of exposed infants in prenatally to opioids(n=1899) was slightly higher than the infants (n=23,011) who were not exposed to opioids (7.1% vs. 6.2%). The adjusted hazard of any neurodevelopmental disorder by DSM among all children exposed to any opioid during any trimester any pregnancy was not significant (HR: 0.92, 95%CI: 0.92,1.32) however the children who were exposed to prolonged opioids and high dose opioids had higher hazard of having any neurodevelopmental disorders compared to those who were not exposed at all. (HR: 1.70, 1.05, 2.76) and (HR: 1.22, 95%CI: 1.22, 1.54). |
| Dubowitz neurological test scores | vanBaar 1989 | Poor | 40 weeks | In utero opioid exposed infants’ (n=29) median score was 1.02 (range:0.67 – 1.55) vs. unexposed infants(n=34) had mean score of 0.94(range:0.54 – 1.72) |
| Einstein object Performance Scale | Johnson 1982 | Fair | 6 months | Exposed infants’ (n=28) mean score was 3.7(1.3) vs. unexposed infants(n=14) had mean score of 3.4 (1.2). |
| Griffiths Mental Development Scale | Bunikowski 1998 | Poor | 9- 17 months | 40.74% of infants exposed to opioids in utero(n=27) had neurological deviations vs. 7.14% of the infants unexposed to opioids in utero(n=42). |
|  | McGlone 2015 | Good | 6 months | The median score among infants exposed to opioids in utero (n=81) was 97 (IQR: 93-100) vs. 105 (IQR: 101-108) among unexposed children(n=26) |
|  | Burns 1996 | Good | 3-7 years | The mean score among preschool children exposed to opioids in utero (n=23) was 110(NA) vs. 107(NA) among unexposed children(n=20) |
| ITPA | Wilson 1979 | Good | 3-6 years | The mean psycholinguistic score among children exposed to opioids in utero (n=22) was 91.4 (NA) vs. 92.0 among unexposed children living in drug environment(n=20) vs. 93.5 unexposed children from medically high-risk pregnancies(n=15) vs. 98.9 children living in average socioeconomic conditions(n=20). |
| Kaufmann Assessment Battery for Children | deCubas 1993 | Good | 6-13 years | Exposed children (n=20) mean score was 98.8 (NA) vs. unexposed children (n=20) had mean score of 102.4 (NA) |
| McCarthy Scales of Children's Abilities | Kaltenbach 1989 | Poor | 3.5-4.5 years | Exposed preschool children (n=27) mean score was 106.51 (12.96) vs. unexposed preschool children (n=17) had mean score of 106.05 (13.1) |
|  | Nygaard 2015 | Fair | 4.5 years | The mean full score among preschool children exposed to opioids in utero (n=71) was 102.33(15.11) vs. 114.3(12.07) among unexposed children(n=54) |
|  | Ornoy 1996^9^ | Fair | 3-6 years | Exposed preschool children (n=35) mean score was 101.7 (13.6) vs. unexposed preschool children from low SES families(n=29) had mean score of 88 (11.4) vs. unexposed healthy preschool children (n=24) had mean score 108.4(12.2). |
|  | Rosen 1985 | Poor | 5 years | Exposed preschool children (n=18) mean score was 89.22 (3.4) vs. unexposed preschool children (n=10) had mean score of 88.9 (3.2) |
|  | Straus 1979 | Poor | 5 years | The mean score in “General Cognitive” domain among preschool children exposed to opioids in utero (n=33) was 86.8(13.3) vs. 86.2(16.2) among unexposed preschool children(n=30).  The mean score in “Memory” domain among preschool children exposed to opioids in utero (n=33) was 43.6(8.2) vs. 44(8.8) among unexposed preschool children(n=30). |
|  | Wilson 1979 | Good | 3-6 years | The mean score in “General Cognitive” domain among preschool children exposed to opioids in utero (n=22) was 88.71(NA) vs. 92.87(NA) among unexposed preschool children raised in drug environment(n=20) vs. 93.08(NA) unexposed children from medically high risk pregnancies (n=15) and 97.42 (NA) from healthy control group(n=20).  The mean score in “Memory” domain among preschool children exposed to opioids in utero (n=22) was 44.31(NA) vs. 48.06(NA) among unexposed preschool children raised in drug environment(n=20) vs. 48.55(NA) unexposed children from medically high risk pregnancies (n=15) and 51.16 (NA) from healthy control group(n=20). |
| NAPLAN test | Oei 2017 | Good | Grade 3 | The mean score in “Reading” domain among children exposed to opioids in utero (n=1663) was 360.8(81.8) vs. 410.3 (86.6) among unexposed children(n=3251).  The mean score in “Numeracy” domain among children exposed to opioids in utero (n=1663) was 350.1(66.5) vs. 393.1 (75.2) among unexposed children (n=3251).  The mean score in “Writing” domain among children exposed to opioids in utero (n=1663) was 365.1 (78.2) vs. 415.3 (69.4) among unexposed children (n=3251).  The mean score in “Grammar” domain among children exposed to opioids in utero (n=1663) was 357.2 (96.8) vs. 417.2 (96.8) among unexposed children (n=3251).  The mean score in “Spelling” domain among children exposed to opioids in utero (n=1663) was 356.5 (82.1) vs. 412.3 (82.3) among unexposed children(n=3251). |
|  |  |  | Grade 5 | The mean score in “Reading” domain among children exposed to opioids in utero (n=1104) was 449.2 (72.9) vs. 490.3 (77.5) among unexposed children(n=2160).  The mean score in “Numeracy” domain among children exposed to opioids in utero (n=1104) was 440.3 (61.6) vs. 485.2 (74.1) among unexposed children(n=2160).  The mean score in “Writing” domain among children exposed to opioids in utero (n=1104) was 428.7 (72.9) vs. 474.8 (67.9) among unexposed children(n=2160).  The mean score in “Grammar” domain among children exposed to opioids in utero (n=1104) was 446.9 (79.9) vs. 496.5 (86.5) among unexposed children(n=2160).  The mean score in “Spelling” domain among children exposed to opioids in utero (n=1104) was 447.3 (79.1) vs. 496.4 (75.1) among unexposed children(n=2160). |
|  |  |  | Grade 7 | The mean score in “Reading” domain among children exposed to opioids in utero (n=499) was 493.5 (68.3) vs. 533.8 (74.7) among unexposed children (n=992).  The mean score in “Numeracy” domain among children exposed to opioids in utero (n=499) was 489.8 (54.4) vs. 536.6 (76.1) among unexposed children(n=992).  The mean score in “Writing” domain among children exposed to opioids in utero (n=499) was 442.4 (100.8) vs. 501.2 (81.3) among unexposed children (n=992).  The mean score in “Grammar” domain among children exposed to opioids in utero (n=499) was 490.7 (77.5) vs. 530.4 (83.7) among unexposed children(n=992).  The mean score in “Spelling” domain among children exposed to opioids in utero (n=499) was 504.2 (81.9) vs. 544.9 (72.6) among unexposed children(n=992). |
| NEPSY | Konijenberg 2016^8^ | Poor | 4 years | The mean score in “Narrative memory” domain among preschool children exposed to opioids in utero (n=35) was 6.91 (3.75) vs. 9.03 (4.32) among unexposed preschool children(n=32).  The mean score in “statue” domain among preschool children exposed to opioids in utero (n=35) was 16.23 (7.86) vs. 22.48 (7.08) among unexposed preschool children(n=32).  The mean score in “Visual Attention” domain among preschool children exposed to opioids in utero (n=35) was 16.8(4.85) vs. 22.48(7.08) among unexposed preschool children(n=32).  The mean score in “Imitating hand positions” domain among preschool children exposed to opioids in utero (n=35) was 9.09(4.07) vs. 12.16(4.35) among unexposed preschool children(n=32). |
| Phelps Kindergarten readiness scale: Total readiness score | Lee 2020 | Good | 4.5 years | Opioid-exposed children’s (n=89) mean score was 82.0 (21.1), unexposed children (n=103) had a mean score of 96.3 (19.3) |
| Referred for Evaluation of Special Education (Percent of Children) | Fill 2018 | Good | 3-8 years old | 5.3 % of exposed children (n=1815) received special education for developmental delays vs. 3.5 % of unexposed children (n=5441).  0.4 % of exposed children (n=1815) received special education for specific learning disability vs. 0.3 % of unexposed children (n=5441).  10.3 % of exposed children (n=1815) received special education for speech or language impairment vs. 8.3 % of unexposed children (n=5441). |
| Reynell Developmental Language Scales | Hunt 2008 | Poor | 38.2 months | The mean score in “Expressive Language” domain among preschool children exposed to opioids in utero (n=67) was 35.5 (7.9) vs. 42.8 (12.6) among unexposed preschool children(n=44).  The mean score in “Comprehension” domain among preschool children exposed to opioids in utero (n=67) was 42.4 (11.6) vs. 49.2(11.4) among unexposed preschool children(n=44). |
| Snijders-Oomen Nonverbal (SON) intelligence test | vanBaar 1994 | Poor | 4 years | The mean score in “Expressive Language” domain among preschool children exposed to opioids in utero (n=26) was 46 (9) vs. 50(6) among unexposed preschool children(n=31).  The mean score in “Comprehension” domain among preschool children exposed to opioids in utero (n=26) was 46 (6) vs. 52(6) among unexposed preschool children(n=32). |
| Speech and Language Assessment Scale (SLAS) | Skovlund 2020 | Good | 5 years | Exposed children’s (n=584) adjusted odds ratio of lower (mean score > 3) vs higher (mean score < 3) than typical for age was 0.84 (0.61, 1.17) compared to unexposed children (n=30,000) |
| Stanford–Binet Intelligence Scales | deCubas 1993 | Good | 6-13 years | Exposed children (n=20) mean score was 97.6 (NA) vs. unexposed children (n=20) had mean score of 98.1(NA) |
|  | Hunt 2008 | Poor | 3 years | Exposed preschool children(n=67) mean score was 99.9 (15.1) vs. unexposed children (n=44) had mean score of 107.5 (13.4) |
|  | Pulsifier 2008 | Good | 5 years | Exposed preschool children(n=113) mean score was 86.7 (11.3) vs. unexposed children (n=31) had mean score of 89.5 (13) |
|  | Nair 2008 | Good | 5 years | Exposed children(n=111) mean score was 88.4 (11) vs. unexposed children(n=62) had mean score of 91.3 (11.2) |
| Twenty Statements about Language-Related Difficulties list (Language 20Q) | Skovlund 2020 | Good | 5 years | Exposed children’s (n=584) adjusted odds ratio of lower (mean score ≥ 2) vs best performance category (mean score 1) scores was 0.57 (0.35,0.91) compared to unexposed children (n=30,000)  Exposed children’s (n=584) adjusted odds ratio of middle (mean score 1.01-1.99) vs best performance category (mean score 1) scores was 1.04 (0.86, 1.26) compared to unexposed children (n=30,000) |
| WASI | Bauer 2020 | Good | 9, 13 years | Exposed children (n=45) had significantly lower verbal IQ scores than unexposed children (n=45) |
| WASI-II | Lee 2019 | Good | 9.5 years | Opioid-exposed children (n=85) had a mean score of 93.83 (14.35), while unexposed children (n=99) had a mean score of 108.33 (13.88) |
| WISC-R/III | Bauer 2020 | Good | 7 years | Exposed children (n=45) had significantly lower verbal IQ scores than unexposed children (n=45) |
|  | Davis 1988 | Good | 6-15 years old children | The mean full-scale IQ in children exposed to opioids in utero (n=28) was 90.36(11.36) vs. 96.32(8.72) among unexposed children(n=28) |
|  | Nygaard 2015^6^ | Fair | 8.5 years | The mean full score among preschool children exposed to opioids in utero (n=53) was 97.9(16) vs. 116.11(14.16) among unexposed children(n=48) |
|  | Ornoy 2001^7^ | Good | 5-12 years | The mean score in “Verbal” domain among preschool children exposed to opioids in utero (n=65) was 105.29(14.35) vs. 110.4(22.1) among average SES unexposed children(n=30)  The mean score in “Performance” domain among preschool children exposed to opioids in utero (n=65) was 103.72(24.42) vs. 115.3(22.4) among unexposed children(n=30) |
|  | Ornoy 2010^7^ | Fair | 12-16 years | The mean score in “Similarities” domain among adolescents exposed to opioids in utero (n=26) was 9.24(1.59) vs. 7.37(2.82) among low SES unexposed children(n=24).  The mean score in “Vocabulary” domain among adolescents exposed to opioids in utero (n=26) was 8.24(2.37) vs. 8(2.67) among low SES unexposed children(n=24).  The mean score in “Arithmatic” domain among adolescents exposed to opioids in utero (n=26) was 7.92(2.08) vs. 8.04(1.74) among low SES unexposed children(n=24).  The mean score in “Picture arrange” domain among adolescents exposed to opioids in utero (n=26) was 8.72(2.92) vs. 8.56(2.21) among low SES unexposed children(n=24).  The mean score in “Block-Design” domain among adolescents exposed to opioids in utero (n=26) was 9.2(3.12) vs. 8.91(3.19) among low SES unexposed children(n=24). |
|  | Walhovd 2010 | Poor | 9 years | The mean IQ score among children exposed to opioids in utero (n=14) was 98.9(9.8) vs. 114.4 (14.9) among unexposed children(n=14) |
| WJ-III (Woodcock Johnson Test of Achievement III) | Bauer 2020 | Good | 7, 8, 10, 12, and 15 years | No differences between exposed and unexposed groups in terms of academic achievement. |
|  | Lee 2019 | Good | 9.5 years | In “Broad Reading” subscale, exposed children (n=84) had a mean score of 87.74 (19.8). Unexposed children (n=99) had a mean score of 104.35 (14.35)  In “Broad Math” subscale, exposed children (n=84) had a mean score of 86.8 (18.37). Unexposed children (n=99) had a mean score of 103.03 (15.42). |
| WPPSI-III/R | Bauer 2020 | Good | 4.5 Years | Opiate exposed preschool children (n=45) had mean scores of 83.5 vs. unexposed children’s (n=45) mean score was 88.3 (p = 0.02) |
|  | Haabrekke 2018 | Poor | 4.5 years | The mean full-scale IQ in preschool children exposed to opioids in utero (n=22) was 95(9.5) vs. 100.4(8.5) among unexposed children(n=26) |
|  | Konijnenberg 2015 | Poor | 4.5 years | The mean score in “Animal Pegs” domain among preschool children exposed to opioids in utero (n=35) was 8.31(3.22) vs. 10.42(2.78) among unexposed children(n=31).  The mean score in “Block Design” domain among preschool children exposed to opioids in utero (n=35) was 8.14(2.14) vs. 9.9 (3.65) among unexposed children(n=31)  The mean score in “Comprehension” domain among preschool children exposed to opioids in utero (n=35) was 10.09(3.24) vs. 11.19(2.97) among unexposed children(n=31)  The mean score in “Sentences” domain among preschool children exposed to opioids in utero (n=35) was 8.29(2.8) vs. 11.61(2.86) among unexposed children(n=31) |
|  | Lee 2020 | Good | 4.5 years | Opioid-exposed children’s (n=89) mean score was 97.9 (15), unexposed children (n=103) had a mean score of 112.2 (14.5) |
|  | Melinder 2013 | Poor | 4 years | The mean score in “picture completion and vocabulary” domain among preschool children exposed to opioids in utero (n=26) was 32.19(5.56) vs. 34.68(5.06) among unexposed children(n=23) |
|  | Walhovd 2015 | Poor | 4.5 years | The mean full score among preschool children exposed to opioids in utero (n=12) was 94.9(7.2) vs. 99.4(8) among unexposed children(n=12) |

Abbreviations: BSID: Bayley Scale for Infant Development; BRIEF-P: Behavior Rating Inventory of Executive Function , Columbia Mental Maturity, ITPA: Illinois Test of Psycholinguistic Abilities; MOUD: Medication for Opioid Use Disorder; MDI: Mental Development Index, NAPLAN: National Assessment Program – Literacy and Numeracy, NEPSY Behavior Rating Inventory of Executive Function, WPPSI-III/WPPSI-R: Wechsler Preschool & Primary Scale of Intelligence; WISC-R: Wechsler Intelligence Scale for Children-III/Revised, WASI/WASI-II: Wechsler Abbreviated Scale of Intelligence/ -II, SES: Socioeconomic Status, IQ: intelligence quotient

^1^ Same cohort as Levine 2018 cognitive measures were measured at the same age, thus Levine 2021 was reported due to larger sample size.

^2^Same cohort as Moe 2002 cognitive measures were measured at the same age, thus Nygaard 2015 was reported due to larger sample size.

^3^Same cohort as Johnson 1982 and Johnson 1985. Thus, only the measures at 18 and 24 months for Rosen 1985 is reported.

^4^Same cohort as VanBaar 1989. Thus, only the measures of VanBaar 1990 are reported.

^5^ Same cohort as Lee 2020. Thus, only the measures of Kim 2021 are reported due to larger sample size.

^6^ Same cohort as Nygaard 2016. Thus, only the measures of Nygaard 2015 are reported.

^7^ Same cohort as Ornoy 2016. Thus, only the measures of Ornoy 2001, Ornoy 2010 are reported.

^8^As Konijenberg 2016, Melinder 2013 and Konijenberg 2013 studies are conducted in the same cohort the NEPSY results at age 4 for Konijenberg 2016 with larger sample size is reported.

^9^ The means and standard deviations were combined for two exposed groups.

^10^ The IQ scores for Reference group was not available for Sandtorv 2018 thus it is not reported in this table.

### Supplemental Table 8: Studies Investigating Prenatal Exposure to Opioids And Behavioral Development

| **Type of Test** | **Study Title** | **Quality Assessment** | **Age of Testing** | **Findings** |
| --- | --- | --- | --- | --- |
| ADHD Rating Scale | Nygaard 2016 | Good | 8 ½ years | Exposed children’s (n=56) mean scores on the ADHD Rating Scale reported by caregiver were 15 (11.4) vs. unexposed children(n=46) had mean scores on the ADHD Rating Scale reported by caregiver of 5.8 (5.1).  Exposed children’s (n=52) mean scores on the ADHD Rating Scale reported by teacher were 12.8 (10.4) vs. unexposed children had mean scores (n= 38) on the ADHD Rating Scale reported by teacher of 6.2 (7). |
|  | Slinning 2004 | Fair | 4 ½ years | Exposed children’s (n=41) mean score on the ADHD- Rating Scale reported by caregiver was 15.2 (9.7) vs. unexposed children (n=49) had a mean score on the ADHD-PC of 8.7 (5.5).  Exposed children’s (n=41) mean score on the ADHD- Rating Scale reported by teacher was 12.9 (10.3) vs. unexposed children (n=49) had a mean score on the ADHD-PC of 5.6 (5.3). |
| ADHD (International classification of diseases) | Garrison-Desany 2022 | Fair | 2-21 years | ADHD diagnosis Cox-Proportional hazard models: opioid exposure was associated with higher risk of ADHD (2.19; 95% CI, 1.10-4.37) after adjustment for covariates. In Penalized Elastic net regression models and interaction of opioids with both cannabis, alcohol and tobacco smoking was associated with increased risks of ADHD by HRs of 1.42 and 1.15, 1.17 respectively. |
| ASSQ (Autism Spectrum Screening Questionnaire) | Sandtorv 2018 | Poor | 6-14 years | Exposed children’s (n=57) mean total ASSQ score was 14.00 (7.98) vs. unexposed children (n=171) had a mean score of 3.57(4.51). |
| Autism (Special Education) | Fill 2018 | Good | 3-8 years | 0.3% of exposed children (n=1815) received special education for Autism vs. 0.4% of unexposed children (n=5441). |
| BRIEF-P  (Behavior Rating Inventory of Executive Function - Preschool version) | Konijnenberg 2015 | Poor | 4 ½ years | Exposed children’s (n=35) mean “inhibition” domain score was 55.97 (11.05) vs. unexposed children (n=31) had mean “inhibition” domain score of 47.42 (7.1).  Exposed children’s (n=35) mean “shifting” domain score was 51.57 (9.39) vs. unexposed children (n=31) had mean “shifting” domain score of 47.13 (8.72).  Exposed children’s (n=35) mean “emotional control” domain score was 51.54 (12.89) vs. unexposed children (n=31) had mean “emotional control” domain score of 49.52 (14.91).  Exposed children’s (n=35) mean “planning” domain score was 55.29 (11.84) vs. unexposed children (n=31) had mean “planning” domain score of 49.06 (9.39).  Exposed children’s (n=35) mean “Global Executive Composite” domain score was 55.94 (12.06) vs. unexposed children (n=31) had mean “Global Executive Composite” domain score of 47.94 (9.37). |
| BNBAS^1^ | Chasnoff 1986^2^ | Fair | 2 days | The mean score of development among all domains on the exposed groups (n=51+13) was 30.84 (7.38) compared to 38.4(5.02) among unexposed groups(n=27). |
|  | Lesser-Katz 1982^3^ | Poor | 3-4 days | The mean score for all BNBAS Behavioral items was 135.02 for exposed infants (n= 10) vs. 132.99 for unexposed infants (n=14). |
|  | Strauss 1975 | Poor | 1 day | Exposed infants mean score was 138.0 (NA) for the sum of all 27 BNBAS Items (n= 22) vs. unexposed infants (n= 22) had mean score of 128.8 (NA) for the sum of all 27 BNBAS Items. |
|  | Van Baar 1989 (2) | Poor | 44 weeks | Exposed children’s median of deviations from optimal score was 1.56 (range: 0.68 – 3.4) for the sum of all BNBAS Items (n=28) vs. unexposed children(n=37) had a median of deviations from optimal score of 1.36 (range: 0.59 – 3.36) for the sum of all BNBAS Items. |
| BSID-II Behavioral Rating Scale (BRS) | Messinger 2004 | Good | 1 years | Exposed infants (n=79) mean score was 43.8 (3.4) vs. unexposed infants (n=949) had mean BRS of 44.3 (1.0). |
|  |  |  | 2 years | Exposed infants (n=80) mean score was 34.4 (3.1) vs. unexposed infants (n=925) had mean BRS of 41.9 (1.0). |
|  |  |  | 3 years | Exposed infants (n=78) mean score was 50.6 (3.6) vs. unexposed infants (n=921) had mean BRS of 56.3 (1.1). |
| BSID III Social-emotional Scale | Salo 2010 | Poor | 3 years | Exposed children’s (n=21) mean Bayley social-emotional scale score was 8.57 (1.53) vs. unexposed children (n=13) had a mean score of 11.08 (2.59). |
| Burks Behavior Rating Scales | Davis 1988 | Good | 6-15 years | Exposed infants (n=28) sum of mean scores was 241.53 vs. unexposed infants (n=28) had mean Burks BRS of 152.8. |
| CBCL (Achenbach) | Bauer 2020 | Good | 3, 5, 7, 9, 11, 13, and 15 years | “Opiate-exposed children (n=45) demonstrated consistently higher problem scores compared to unexposed children (n=45) for total (p<0.0176), externalizing (p<0.02), and internalizing (p<0.02) behaviors compared to unexposed |
|  | Haabrekke 2018 | Poor | 4 ½ years | Exposed children’s (n=22) mean CBCL total score was 31.9 (27.9) vs. unexposed children (n=26) had mean CBCL total score of 15.5 (10.9).  The mean score for “externalizing behaviors” domain was 11.3 (9.5) for exposed children (n=22) and 6.6 (4.7) for unexposed children (n=26).  The mean score for “internalizing behaviors” domain was 9.5 (9.2) for exposed children (n=22) and 3.4 (3.3) for unexposed children (n=26). |
|  | Konijnenberg 2015 (2) | Poor | 4 ½ years | Exposed children’s (Methadone) (n=22) mean CBCL attention problems score was 54 (5.67), exposed children’s (Buprenorphine) (n=9) mean CBCL attention problems score was 52 (2.29) vs. unexposed children (n=25) had mean CBCL attention problems score of 50.64 (1.8).  The average SNP effect in children exposed to methadone in utero (n=22) was -12(85.93) vs. children exposed to Buprenorphine in utero(n=9) was 5.78(91.39) compared to comparison group of unexposed children(n=25) was 23.5 (45.5). |
|  | Melinder 2013 | Poor | 4 years | Exposed children’s (n=26) mean CBCL “attention problems” domain score was 53.92 (5.27) vs. unexposed children (n=23) had mean CBCL “attention problems” domain score of 50.68 (1.92). |
|  | Nair 2008 | Good | 7 years | Exposed children’s (n=111) mean “internalizing problems” domain score was 49.8 (10.2) vs. unexposed children (n=62) had mean “internalizing problems” domain score of 49.4 (9.3).  Exposed children’s (n=111) mean “externalizing problems” domain score was 51 (10.8) vs. unexposed children (n=62) had mean “externalizing problems” domain score of 47.6 (8).  Exposed children’s (n=111) mean “total behavior problems” domain score was 50.7(10.4) vs. unexposed children (n=62) had mean “total behavior problems” domain score of 48.3 (8.8). |
|  | Nygaard 2016 | Good | 8 ½ years | Exposed children’s (n=57) mean “internalizing” domain score was 7.4 (4.1) vs. unexposed children (n=47) had mean “internalizing” domain score of 4.1 (4.3).  Exposed children’s (n=57) mean “externalizing” domain score was 11.3 (10.3) vs. unexposed children (n=47) had mean “externalizing” domain score of 4.8 (6.4).  Exposed children’s (n=57) mean “social problems” domain score was 2.6 (2.8) vs. unexposed children (n=47) had mean “social problems” domain score of 0.9 (1.6).  Exposed children’s (n=57) mean “attention problems” domain score was 5.1 (4.2) vs. unexposed children (n=47) had mean “attention problems” domain score of 1.7 (2.3). |
|  | Ornoy 2001 | Good | 5 – 12 years | Exposed children’s (at home, n=31) mean “externalizing problems” domain score was 20.07 (13.5), and exposed children’s (adopted, n=34) mean “externalizing problems” domain score was 13.50 (9.13) vs. unexposed children (drug-dependent fathers, n=33) had “externalizing” domain score of 16.41 (9.05), unexposed children (low SES) had “externalizing” domain score of 12.77 (9.48), and unexposed children (average SES) had “externalizing” domain score of 3.6 (4.01).  Exposed children’s (at home, n=31) mean “internalizing problems” domain score was 9.16 (4.94), and exposed children’s (adopted, n=34) mean “internalizing problems” domain score was 5.88 (4.99) vs. unexposed children (drug-dependent fathers, n=33) had “internalizing” domain score of 7.87 (5.67), unexposed children (low SES) had “internalizing problems” domain score of 9.13 (8.46), and unexposed children (average SES) had “internalizing problems” domain score of 3.7 (5.17). |
|  | Ornoy 2010 | Fair | 12 – 16 years | Exposed children’s (low SES, n=26) mean CBCL total problems’ score was 58.52 (11.78), and exposed children’s (adopted, high SES, n=29) mean CBCL total problems’ score was 58.75 (9.28) vs. unexposed children (low SES, n=24) had mean CBCL total score of 58.30 (11.27), and unexposed children (high SES, n=23) had mean CBCL total score of 51.38 (11.99).  Exposed children’s (low SES, n=26) mean “internalizing problems” domain score was 59.16 (11.77), and exposed children’s (adopted, high SES, n=29) mean “internalizing problems” domain score was 58.00 (9.41) vs. unexposed children (low SES, n=24) had mean “internalizing problems” domain score of 58.00 (9.69), and unexposed children (high SES, n=23) had mean “internalizing problems” domain score of 52.26 (10.31).  Exposed children’s (low SES, n=26) mean “externalizing problems” domain score was 56.04 (13.72), and exposed children’s (adopted, high SES, n=29) mean “externalizing problems” domain score was 56.75 (14.31) vs. unexposed children (low SES, n=24) had mean “externalizing problems” domain score of 55.37 (9.94), and unexposed children (high SES, n=23) had mean “externalizing problems” domain score of 49.84 (11.36). |
|  | Ornoy 2016 | Poor | 5 – 16 ½ years | Exposed children’s (n=38) mean “internalizing problems” domain score was 9.6 (7.5) vs. unexposed children (n=46) had mean “internalizing problems” domain score of 9.2 (8.4).  Exposed children’s (n=38) mean “externalizing problems” domain score was 13.8 (9.5) vs. unexposed children (n=46) had mean “externalizing problems” domain score of 11.6 (8.7). |
|  | Sarfi 2013 | Poor | 2 ½ years | Exposed children’s (n=33) mean CBCL total problems score was 28.2 (15.9) vs. unexposed children (n=35) had mean CBCL total problems score of 16.9 (10.7). |
|  | Slinning 2004 | Fair | 2 years | Exposed children’s (n=42) mean “attention problems” domain score was 3 (2) vs. unexposed children (n=50) had mean “attention problems” domain score of 1.8 (1.6). |
|  |  |  | 4 ½ years | Exposed children’s (n=42) mean “attention problems” domain score was 3.4 (2.9) vs. unexposed children (n=50) had mean “attention problems” domain score of 1.8 (1.6). |
|  | deCubas 1993 | Good | 6-13 years | Exposed children’s (n=20) mean “internalizing problems” domain score was 59.2 vs. unexposed children (n=20) had mean “internalizing problems” domain score of 46.3.  Exposed children’s (n=20) mean “externalizing problems” domain score was 60.3 vs. unexposed children (n=20) had mean “externalizing problems” domain score of 48.6. |
| Conner’s Questionnaire | Ornoy 2001 | Good | 5 – 12 years | Exposed children’s (at home, n=31) mean score was 19.89 (10.14) vs. exposed children’s (adopted, n=34) mean score was 14.47 (10.02) vs. unexposed children (drug-dependent fathers, n=33) had a score of 16.03 (8.88) vs. unexposed children (low SES) had a score of 11.57 (10.68) vs. unexposed children (average SES) had a score of 5.14 (6.3). |
|  | Ornoy 2016 | Poor | 5 – 16 ½ years | Exposed children’s (n=38) average score was 58.2 (11.9) vs. unexposed children (n=46) had a mean score of 55.1 (11.5). |
|  | Trønnes2021 | Good | 11 years old | No association between exposure to opioid analgesic use in separate periods in pregnancy and ADHD diagnosis or prescription rates by pregnancy. The 95% CI for the adjusted HR was wide and included the null values. However, there was an association between >5 weeks of opioid analgesic use and increased risk of ADHD compared to ≤4weeks use. (HR=1.60; 95%CI 1.04, 2.47) |
| Emotional Availability Scales | Salo 2010 | Poor | 8-12 months | Exposed children’s (n=15) mean emotional availability scale “infant responsiveness” domain scores were 4.26 (1.29) vs. unexposed children (n=57) had mean “infant responsiveness” domain scores of 5.72 (0.79).  Exposed children’s (n=15) mean emotional availability scale “infant involvement” domain scores were 3.87 (1.25) vs. unexposed children (n=57) had mean “infant involvement” domain scores of 5.69 (0.84). |
|  | Salo 2009 | Poor | 3 years | Exposed children’s (n=21) mean emotional availability scale “infant responsiveness” domain scores were 4.55 (0.92) vs. unexposed children (n=13) had mean “infant responsiveness” domain scores of 6 (0.54).  Exposed children’s (n=21) mean emotional availability scale “infant involvement” domain scores were 4.5 (0.89) vs. unexposed children (n=13) had mean “infant involvement” domain scores of 5.96 (0.51). |
| Emotional/Behavioral Disorder | Hall 2019 | Poor | >24 months | Out of children exposed to opioids without developing NAS after birth (n=473), 3% were diagnosed with behavioral or emotional disorder. 5.8% of exposed children with NAS after birth (n=138) were diagnosed with behavioral or emotional disorder. In unexposed children (n=14,933), 1.1% were diagnosed with behavioral or emotional disorder. |
| Emotional Disorder/ Conduct disorder | Azuine 2019 | Fair | >6 years | Exposed children’s (n=2391) odds of conduct disorder or emotional disorder diagnosis was 1.79 (95% CI: 0.9, 3.35) compared to unexposed children (n=2391).  The adjusted odds of being diagnosed with ADHD among children ≥6 years old was 2.55 (95%CI: 1.42.-4.57). |
|  |  |  | <6 years | Exposed children’s (n=3106) odds of diagnosis of conduct disorder or emotional disorder was 2.137 (95% CI: 1.2, 3.77) compared to nonexposed children (n=3106).  The adjusted odds of being diagnosed with ADHD among children < 6 years old was 1.01(95%CI: 0.46.-2.23). |
| Fantz/Newis Visual habituation task | Johnson 1982 | Fair | 6 months | Exposed infant’s (n=28) mean score was 8.3 (27.7) vs. unexposed infants (n=15) had a mean score of -4.8 (21.6). |
| Infant Behavior Record | Van Baar 1994 | Poor | 3 ½ years | Exposed children’s (n=22) median free of fear subdomain score were 9 (range: 4 – 9) vs. unexposed children (n=32) had a median score of 6.5 (range 2 – 9).  Exposed children’s (n=22) median IBR attention subdomain span scores were 5 (range: 1 – 7) vs. unexposed children (n=32) had a median score of 5.5 (range 1 – 9).  Exposed children’s (n=22) median IBR activity level subdomain score was 6 (range: 3 – 9) vs. unexposed children (n=32) had a median score of 5 (range 2 – 9). |
|  |  |  | 4 ½ years | Exposed children’s (n=23) median IBR attention subdomain span scores were 5 (range: 2 – 8) vs. unexposed children (n=31) had a median score of 5 (range: 2 – 8).  Exposed children’s (n=23) median IBR endurance subdomain score was 4 (range: 2 – 9) vs. unexposed children (n=31) had a median score of 6 (range 1 – 9).  Exposed children’s (n=23) median IBR cooperation subdomain score was 6 (range: 2 – 9) vs. unexposed children (n=31) had a median score of 7 (range 3 – 9). |
|  |  |  | 5 ½ years | Exposed children’s (n=22) median IBR free of fear subdomain scores were 8 (range 2 – 9) vs. unexposed children (n=30) had a median score of 9 (range 5 – 9).  Exposed children’s (n=22) median IBR attention subdomain span scores were 5 (range 2 – 8) vs. unexposed children (n=30) had a median score of 5 (range 3 – 9).  Exposed children’s (n=22) median IBR cooperation subdomain score was 6 (range 1 to 9) vs. unexposed children (n=30) had a median score of 8 (range 4 – 9). |
|  | Wilson 1981 | Poor | 9 months | 29% of exposed children (untreated, n=29) showed short attention span and 24% of exposed children (Methadone, n=35) showed short attention span vs. 7% of unexposed children (n=55) showed short attention span. |
|  | Hans 2001 | Good | 4 months | Exposed children’s (n=33) mean IBR “activity level” domain score was 7.9 (1.8) vs. unexposed children (n=45) had mean score of 7 (1.4).  Exposed infant’s (n=33) mean IBR “attention sum” domain score was 12.8 (3.9) vs. unexposed infants (n=45) had mean score of 13.1 (4.2). |
|  |  |  | 8 months | Exposed children’s (n=33) mean IBR “activity level” domain score was 8.4 (1.8) vs. unexposed children (n=45) had mean score of 8.1 (1.5).  Exposed infant’s (n=33) mean IBR “attention sum” domain score was 17.7 (2.1) vs. unexposed infants (n=45) had mean score of 18 (2.6). |
|  |  |  | 12 months | Exposed children’s (n=33) mean IBR “activity level” domain score was 8.6 (1.3) vs. unexposed children (n=45) had mean score of 8.6 (1.7).  Exposed infant’s (n=33) mean IBR “attention sum” score was 16.3 (2.8) vs. unexposed infants (n=45) had mean score of 18.2 (2.7). |
|  |  |  | 18 months | Exposed children’s (n=33) mean IBR “activity level” domain score was 8.9 (1.5) vs. unexposed children (n=45) had mean score of 8.9 (1.8).  Exposed infant’s (n=33) mean IBR “attention sum” score was 16.8 (2.5) vs. unexposed infants (n=45) had mean score of 17.4 (2.5). |
|  |  |  | 24 months | Exposed children’s (n=33) mean IBR “activity level” domain score was 9.2 (1.5) vs. unexposed children (n=45) had mean score of 8.7 (1.7).  Exposed infant’s (n=33) mean IBR “attention sum” score was 16.5 (3.1) vs. unexposed infants (n=45) had mean score of 17.4 (2.5). |
|  |  |  | 4-24 months | Exposed children’s (n=33) mean IBR “activity level” domain score was 8.6 (1.0) vs. unexposed children (n=45) had mean score of 8.3 (1.0).  Exposed infant’s (n=33) mean IBR “attention sum” score was 16 (1.4) vs. unexposed infants (n=45) had mean score of 16.8 (1.6). |
| The Infant Behavior Questionnaire-Revised (IBQ-R) | Bakhireva 2019 | Poor | 5-8 months | Exposed infants’ (n=42) mean IBQ-R “negative affect” domain score was 4.23 (1.13) vs. unexposed infants (n=36) had mean IBQ-R “negative affect” domain score of 4 (1.1).  Exposed infants’ (n=42) mean IBQ-R “effortful control” domain score was 5.8 (2.3) vs. unexposed infants (n=36) had mean IBQ-R “effortful control” domain score of 5.6 (2.4).  Exposed infants’ (n=42) mean IBQ-R “surgency” domain score was 5.5 (2.3) vs. unexposed infants (n=36) had mean IBQ-R “surgency” domain score of 5.1 (2.8). |
| Infants’ performance on Interaction | Bernstein 1984 | Poor | 4 months | Exposed children’s (n=17) mean performance on interaction score was 15.41 (2.24) vs. unexposed children (n=23) had a mean score of 14.26 (2.24). |
| Infant-Toddler Symptom Checklist | Levine 2021 | Good | 24 months | The children in opioid exposed group had higher average total dysregulation score 10.69 (10.33) compared to unexposed children 4.95 (5.69). Linear Regression models suggested that the children exposed to opioids had higher total dysregulation score compared to unexposed children after adjusting for confounders (b=0.56 95%CI:0.14,0.97) |
| IOWA Gambling Task- Child Version | Konijnenberg 2021 | Poor | 11 years | The children in the opioid medication therapy (OMT) group exhibited good decision-making abilities than the control group. OMT group were more likely to avoid doors with the high punishment than the children in comparison group. The children in healthy control group selected advantageous decision faster than the OMT group. |
| McGuire-Richman Pre-School Behavior Checklist/ Mertin Herbet: Child Care and Family - Checklist | Burns 1996 | Good | 3-7 years | Exposed children and unexposed children showed similar results in the questionnaires. |
| Mother-Child interaction (sum) | Konijnenberg 2016 | Poor | 1 year | Generally, mother-child interactions in the exposed group were rated less positive than the unexposed group in terms of interactions with mothers. Exposed children’s (n=35) mean sum of the mother-child interaction score was 62.24 vs. unexposed children (n=32) had a mean score of 69.98. |
|  |  |  | 4 years | Exposed children’s (n=35) mean sum of the mother-child interaction score was 85.95 vs. unexposed children (n=32) had a mean score of 104.51. |
| NICU Network Neurobehavioral Scale (NNNS) | Bauer 2020 | Good | 1 month | No differences between exposed (n=45) and unexposed (n=45) children were observed |
|  | Lester 2002^4^ | Good | 1 month | Exposed children’s (n= 91) mean sum score NNNS elements was 38.38 (0.49) vs. unexposed children (n= 1120) had an adjusted mean sum score of 36.54 (0.16). |
|  | Wouldes 2020 | Good | 1 week | Exposed children’s (n= 71-86) adjusted mean sum score of NNNS elements was 33.27(4.53) vs. unexposed children (n= 98-103) had a mean sum score of 32.55(3.73). |
| Pediatric Quality of Life | Sarfi 2013 | Poor | 2 ½ years | Exposed children’s (n=33) mean PedQL total score was 84.6 (10.1) vs. unexposed children (n=35) had mean PedQL total score of 91.5 (7.6). |
| Pollack Test | Ornoy 2001 | Good | 5 – 12 years | Exposed children’s (at home, n=31) mean Pollack test score was 29.25 (10.07) vs. exposed children’s (adopted, n=34) mean score was 33.08 (6.45) vs. unexposed children (drug-dependent fathers, n=33) had a score of 29.77 (7.07) vs. unexposed children (low SES) had a score of 33.86 (5.36) vs. unexposed children (average SES) had a score of 33.21 (7.66). |
| Roberts apperception test for children | deCubas 1993^5^ | Good | 6-13 years | Exposed children’s (n=20) mean sum score in all domains was 611.2 (SD=NA) vs. unexposed children (n=20) had a mean sum score of 567.4(SD=NA). |
| Strengths and Difficulties Questionnaire (SDQ), Total difficulties score | Lee 2020 | Good | 4.5 years | Opioid-exposed children’s (n=89) mean score was 10.3 (13.1), unexposed children (n=103) had a mean score of 5.6 (4.1). |
|  | Jaekel 2021^6^ | Good | 2 years  4.5 years  9 years | The mean difference of total difficulties test scores among opioid exposed children was significantly higher compared to unexposed children (2.06, 95%CI: 0.61,3.52).  The mean difference of total difficulties test scores among opioid exposed children was significantly higher compared to unexposed children (7.13, 95%CI: 5.30, 8.96)  Opioid exposed children exhibited overall higher difficulties scores which tended to worsen by age (b of sex* opioid interaction=2.40, 95%CI:1.46, 3.34)) |
|  | Sarfi 2022 | Poor | 11 years | The mean score for total difficulties was lower among children exposed to OMT and raised at home (n=60) compared to foster OMT children raised at foster care (n=16) and unexposed children raised at foster care (n=140) (Mean=8.5;16.3 and 14.9) (SD=5.6;6.3 and 7.8) |
| Snack Delay Task: inhibitory control | Levine 2018 | Poor | 2 years | Exposed infant’s (n=61) mean snack delay task: inhibitory control score was 7.34 (2.97) vs. unexposed infants (n=82) had a mean score of 9.44 (2.86). |
| SNAP-IV (ADHD screening tool) combined | Sandtorv 2018 | Poor | 6-14 years | Exposed children’s (n=57) mean combined SNAP score was 19.65 (9.06) vs. unexposed children (n=171) had a mean score of 4.13 (5.37). |
| Still Face Paradigm (SFP): Infant positive effect | Lowe 2017 |  | 6 months | There was a non-significant inverse association between being exposed to opioids in utero and infant positive effect (b= – 0.2) |
| Teachers Report Form (TRF) | Haabrekke 2018 | Poor | 4 ½ years | Exposed children’s (n=22) mean TRF total score was 19.7 (19.5) vs. unexposed children (n=26) had mean TRF total score of 24 (21).  Exposed children’s (n=22) mean “externalizing problems” domain score was 9.4 (12.2) vs. unexposed children (n=26) had mean “externalizing problems” domain score of 48.6.  Exposed children’s (n=22) mean “internalizing problems” domain score was 12.1 (13.6) vs. unexposed children (n=26) had mean “internalizing problems” domain score of 6 (5.3). |
|  | Nygaard 2016 | Good | 8 ½ years | Exposed children’s (n=60) mean “internalizing problems” domain score was 6.6 (7) vs. unexposed children (n=42) had mean “internalizing problems” domain score of 3.9 (6.5).  Exposed children’s (n=60) mean “externalizing problems” domain score was 9.7 (12) vs. unexposed children (n=42) had mean “externalizing problems” domain score of 48.6.  Exposed children’s (n=60) mean “attention problems” domain score was 9.2 (8.5) vs. unexposed children (n=42) had mean “attention problems” domain score of 4.2 (4.9). |
|  | Slinning 2004 | Fair | 4 ½ years | Exposed children’s (n=42) mean score on the TRF was 7.3 (6) vs. unexposed children (n=50) had a mean score of 2.4 (2.7). |
| VABS (Vineland Adaptive Behavior Scales) | Bauer 2020 | Good | 3, 6, and 10 years | Exposed (n=45) and unexposed (n=45) group did not differ in any of the four domains assessed. |
| Vineland Social Maturity Scale | Hunt 2008 | Poor | 1 ½ years | Exposed children’s (n=79) mean Vineland Social Maturity Scale score was 113.2 (15.6) vs. unexposed children (n=61) had a score of 119.15 (17.5). |
|  |  |  | 3 years | Exposed children’s (n=67) mean Vineland Social Maturity Scale score was 38.4 (8.1) vs. unexposed children (n=44) had a score of 46.1 (7.7). |
| Werry-Weiss-Peters Activity Questionnaire (Hyperactivity) | Van Baar 1990 | Poor | 18 months | Exposed infants (n=17) median score was 1.76 (range: 1.40 – 2.62) vs. unexposed infants (n=30) had a median activity questionnaire result of 1.73 (range: 1.23 – 2.52). |
|  |  |  | 24 months | Exposed infants (n=21) median score was 1.71 (range: 1.33 – 2.60) vs. unexposed infants (n=31) had a median activity questionnaire result of 1.76 (range: 1.19 – 2.52). |
|  |  |  | 30 months | Exposed infants’ (n=19) median score was 1.62 (1.03 – 2.66) vs. unexposed infants (n=30) had a median activity questionnaire result of 1.64 (range: 1.28 – 2.52). |
| Youth Self Report (YSR) | Ornoy 2010 | Fair | 12 – 16 years | Exposed children’s (low SES, n=26) mean YSR total score was 49.44 (9.08) (Total), and exposed children’s (adopted, high SES, n=29) mean YSR total score was 46.00 (9.06) vs. unexposed children (low SES, n=24) had mean YSR total score of 51.19 (9.34), and unexposed children (high SES, n=23) had mean YSR total score of 49.62 (8.69).  Exposed children’s (low SES, n=26) mean “internalizing problems” domain score was 47.60 (9.79), and exposed children’s (adopted, high SES, n=29) mean “internalizing problems” domain score was 45.79 (9.21) vs. unexposed children (low SES, n=24) had mean “internalizing problems” domain score of 50.44 (13.02), and unexposed children (high SES, n=23) had mean “internalizing problems” domain score of 50.16 (8.24).  Exposed children’s (low SES, n=26) mean “externalizing problems” domain score was 52.36 (9.01), and exposed children’s (adopted, high SES, n=29) mean “externalizing problems” domain score was 47.57 (10.40) vs. unexposed children (low SES, n=24) had mean “externalizing problems” domain score of 47.85 (13.25), and unexposed children (high SES, n=23) had mean “externalizing problems” domain score of 50.32 (9.51). |

ADHD: Attention Deficit Hyperactivity Discorder, ASSQ: Autism Spectrum Screening Questionnaire, BNBAS: Brazelton Neonatal Behavioral Assessment Scale (BNBAS), BSID: Bayley Scales for Infant Development, BRS: Behavioral Rating Scale, BRIEF-P: Behavior Rating Inventory of Executive Function - Preschool version, CBCL: Child Behavior Checklist, IBR: Infant Behavior Record, OMT: Opioid maintenance treatment, PedQL: Pediatric Quality of Life, SES: Socioeconomic Status, TRF: Teachers Report Form, VABS: Vineland Adaptive Behavior Scales, Youth Self Report: (YSR)

^1^ Different studies selected different items from BNBAS instrument to study newborn and infants’ neurodevelopment.

^2^We calculated the average and standard deviation of sum of all BNBAS items based on the findings reported in the study. We also combined the two exposed groups in this study to report means and standard deviations.

^3^We calculated the average and standard deviation of sum of all BNBAS items based on the findings reported in the study.

^4^We calculated the average and standard deviation of sum of all NICU Network Neurobehavioral Scale (NNNS) items based on the findings reported in the study.

^5^We calculated the average and standard deviation of sum of all Roberts apperception test for children items based on the findings reported in the study.

^6^Same cohort as Lee 2020. The average SDQ scores for Lee 2020 for the age 4.5 years were reported due to higher sample size.

### Supplemental Table 9: Studies Investigating Prenatal Exposure to Opioids and Motor Development

| **Type of Test** | **Study Title** | **Quality Assessment** | **Age of Testing** | **Findings** |
| --- | --- | --- | --- | --- |
| Basal/Adaptive esophageal motility: Swallow propagation | Hart 2019 | Poor | 4-9 weeks | Exposed infant’s (n=6) basal esophageal motility: swallow propagation was complete in 73.3%, incomplete in 23.3%, and failed in 3.3% of infants vs. unexposed infants (n=12) basal esophageal motility: swallow propagation was complete in 81.5%, incomplete in 12.6%, and failed in 5.9% of infants.  Exposed infant’s (n=6) adaptive esophageal motility: swallow propagation was complete in 81.9%, incomplete in 9.4%, and failed in 9.4% of infants vs. unexposed infants (n=12) adaptive esophageal motility: swallow propagation was complete in 91%, incomplete in 5%, and failed in 4% of infants. |
| Bayley Infant Behavior Record: Sum Score of five motor Items | Marcus 1982 | Poor | 4 months | Exposed infant’s (n=15) sum score of five motor items from the Bayley Infant Behavior Record was 22.20 (2.57) vs. unexposed infants (n=23) had a score of 18.13 (2.72). |
| BSID PDI | Chasnoff 1986 | Fair | 3 months | Exposed infants (n=36) mean score was 104.3 (11.8) vs. unexposed infants (n=34) had mean of 102.8 (7.0). |
|  |  |  | 6 months | Exposed infants (n=36) mean score was 102.2 (11.9) vs. unexposed infants (n=34) had mean of 107.6 (15.1). |
|  |  |  | 12 months | Exposed infants (n=36) mean score was 104.4 (11.9) vs. unexposed infants (n=34) had mean of 103.4 (12.5). |
|  |  |  | 24 months | Exposed infants (n=36) mean score was 100.3 (14.2) vs. unexposed infants (n=34) had mean of 98.2 (9.8). |
|  | Van Baar 1990^1^ | Poor | 6 months | Exposed infants (n=27) mean score was 118 (18) vs. unexposed infants (n=37) had mean of 114 (21). |
|  |  |  | 12 months | Exposed infants (n=26) mean score was 111 (20) vs. unexposed infants (n=34) had mean of 119 (20). |
|  |  |  | 18 months | Exposed infants (n=22) mean score was 110 (19) vs. unexposed infants (n=33) had mean of 112 (19). |
|  |  |  | 24 months | Exposed infants (n=26) mean score was 105 (17) vs. unexposed infants (n=34) had mean of 100 (18). |
|  |  |  | 30 months | Exposed infants (n=25) mean score was 98 (19) vs. unexposed infants (n=34) had mean of 101 (24). |
|  | Ornoy 1996 | Fair | ½-2 Years | Exposed infants (n=30) mean score was 96.6 (13.3) vs. unexposed children (low SES, neglect) (n=18) had mean Bayley PDI score of 84.4 (12.9) and unexposed children (n=47) had mean Bayley PDI score of 100.9 (14.5). ( |
|  | Messinger 2004 | Good | 1 year | Exposed infants (n=79) mean score was 88.9 (1.6) vs. unexposed infants (n=939) had mean of 90 (0.4). |
|  |  |  | 2 years | Exposed infants (n=76) mean score was 89.0 (1.7) vs. unexposed infants (n=859) had mean of 95.2 (0.5). |
|  |  |  | 3 years | Exposed infants (n=75) mean score was 89.2 (1.6) vs. unexposed infants (n=866) had mean of 93.4 (0.5). |
|  | Hans 2001 | Good | 4 months | Exposed infant’s (n=33) mean PDI score was 116 (12.5) vs. unexposed infants (n=45) had mean score of 121 (12.3). |
|  |  |  | 8 months | Exposed infant’s (n=33) mean PDI score was 111 (12.4) vs. unexposed infants (n=45) had mean score of 111 (12.4). |
|  |  |  | 12 months | Exposed infant’s (n=33) mean PDI score was 106 (18.0) vs. unexposed infants (n=45) had mean score of 110 (17.7). |
|  |  |  | 18 months | Exposed infant’s (n=33) mean PDI score was 105 (17.6) vs. unexposed infants (n=45) had mean score of 109 (17.8). |
|  |  |  | 24 months | Exposed infant’s (n=33) mean PDI score was 100 (14.2) vs. unexposed infants (n=45) had mean score of 108 (14.9). |
|  |  |  | 4-24 months | Exposed infant’s (n=33) mean PDI score was 108 (9.2) vs. unexposed infants (n=45) had mean score of 112 (10.4). |
|  | Wilson 1981 | Poor | 9 months | Exposed infant’s (untreated, n=29) mean score was 92.2 (19.2) vs. exposed infant’s (Methadone, n=35) mean score was 89.9 (12.6) vs. unexposed infants (n=55) had mean PDI score of 99 (14.5). |
|  | Bernstein 1984 | Poor | 4 months | Exposed infant’s (n=17) mean score was 116.6 (15.1) vs. unexposed infants (n=23) had mean PDI score of 118 (10.6). |
|  | Levine 2018 | Poor | 2 years | Exposed children’s (n=68) mean score was 82.94 (20.54) vs. unexposed children (n=88) had mean PDI score of 96.1 (16.38). |
|  | Hunt 2008 | Poor | 19.9 months | Exposed infant’s (n=79) PDI score was 107.5 (16.8) vs. unexposed infants (n=61) had a score of 110.13 (14.7). |
|  | Johnson 1982 | Fair | 6 months | Exposed infant’s (n=39) PDI score was 101 (18.2) vs. unexposed infants (n=23) had a score of 105.1 (14.2). |
|  | Johnson 1985 | Fair | 1 year | Exposed infants (n=46) mean score was 94.8 (SE: 2.53) vs. unexposed infants (n=22) had mean of 102.7 (SE: 2.3). |
|  |  |  | 2 years | Exposed infants (n=46) mean score was 99.1 vs. unexposed infants (n=22) had mean of 108.4. |
|  | Moe 2002 | Fair | 1 year | Exposed infant’s (n=64) PDI score was 82.8 (15.5) vs. unexposed infants (n=52) had a score of 90.8 (10.6). |
|  | Bauer 2020 | Good | 1-3 years | No group differences. |
|  | Rosen 1985 | Poor | 6 months | Exposed infants (n=41) mean score was 105.0 (SE: 2.8) vs. unexposed infants (n=23) had mean Of 105.1 (SE: 2.9). |
|  |  |  | 12 months |  |
|  |  |  | 18 months | Exposed infants (n=38) mean score was 92.6 (SE: 2.4) vs. unexposed infants (n=23) had mean Of 105.3 (SE: 2.2). |
|  |  |  | 24 months | Exposed infants (n=34) mean score was 99.1 (SE: 2.7) vs. unexposed infants (n=22) had mean of 108.0 (SE: 2.7). |
|  | Strauss 1976^2^ | Poor | 3 months | Exposed infant’s (n=25) mean PDI score was 119.4 (9.1) vs. unexposed infants (n=26) had mean score of 117.1 (14.5). |
|  |  |  | 6 months | Exposed infant’s (n=25) mean PDI score was 109.4 (12.2) vs. unexposed infants (n=26) had mean score of 111.7 (14.5). |
|  | Strauss 1979 | Poor | 1 years | Exposed infant’s (n=33) PDI score was 103.3 (13.1) vs. unexposed infants (n=30) had a score of 110.1 (9.3). |
| BSID-II PDI | Levine 2021 | Good | 2 years | Methadone exposed children (n=92) had lower average PDI scores 82.78 (20.46) compared to unexposed children (n=108) 95.20 (15.66) Additionally the proportion of exposed children with motor delays (≤1SD below comparison group mean PDI score) was significantly higher compared to unexposed children (32.5% vs. 13%). Linear regression models suggested that the children exposed to opioids had lower motor development scores (PDI) compared to unexposed children (b=−9.16; 95%CI: −16.51, −1.80) |
|  | Robbins 2021 | Good | 2 years | No significant association between opioid use during labor and odds of any motor delay (OR=0.1.02, 95%CI: 0.80, 1.28) Results were unchanged when stratified by the severity of delay. |
| BSID-III: Motor development domain | Bakhireva 2019 | Poor | 5-8 months | Exposed children’s (n=42) mean score was 95.97 (9.67) vs. unexposed children (n=36) had mean BSID-III: Motor score of 95.6 (12.9). |
|  | Beckwith 2015 | Poor | 1-42 months | Exposed children’s (n=28) mean score was 96.25 (8.64) vs. unexposed children (n=1700) had mean score of 100 (15). |
| Bender Gestalt II fine motor | Konijnenberg 2013 | Poor | 4 ½ years | Exposed children’s (n=15) mean Bender Gestalt II fine motor score was 4.20 (2.18) vs. unexposed children (n=15) had a mean score of 7.33 (2.55). |
| Berry-Buktenica Developmental Test of Visual-Motor Integration | Lee 2020 | Good | 4.5 years | Opioid-exposed children’s (n=89) mean score was 91.8 (13.1), unexposed children (n=103) had a mean score of 102.2 (12.9) |
| Developmental Test of Visual-Motor Integration (VMI-4) | Pulsifer 2008 | Good | 5 years | Exposed children’s (n=113) mean score on the VMI-4 was 88.6 (12.5) vs. unexposed children (n=31) had a mean score of 89.1 (19.3). |
| Griffiths scale of infants' development: psychomotor retardation | Bunikowski 1998 | Poor | 9-17 months | 7.41% of exposed infants (n=27), showed severe psychomotor retardation and 25.92% showed mild psychomotor retardation vs. of unexposed infants (n=42), 0% showed severe psychomotor delays and 7.1% showed mild psychomotor retardation. |
| Infant Behavior Record: Coordination Problems | Hans 2001 | Good | 4 months | Exposed children’s (n=33) mean IBR “coordination problems” domain score was 6.0 (1.1) vs. unexposed children (n=45) had mean score of 5.8 (1.1). |
|  |  |  | 8 months | Exposed children’s (n=33) mean IBR “coordination problems” domain score was 6.1 (1.2) vs. unexposed children (n=45) had mean score of 5.6 (1.2). |
|  |  |  | 12 months | Exposed children’s (n=33) mean IBR “coordination problems” domain score was 5.8 (1.2) vs. unexposed children (n=45) had mean score of 5.5 (0.9). |
|  |  |  | 18 months | Exposed children’s (n=33) mean IBR “coordination problems” domain score was 5.7 (1.0) vs. unexposed children (n=45) had mean score of 5.4 (1.1). |
|  |  |  | 24 months | Exposed children’s (n=33) mean IBR “coordination problems” domain score was 5.5 (1.2) vs. unexposed children (n=45) had mean score of 4.7 (1.3). |
|  |  |  | 4-24 months | Exposed children’s (n=33) mean IBR “coordination problems” domain score was 5.8 (0.7) vs. unexposed children (n=45) had mean score of 5.4 (0.7). |
| Infant Behavior Record: Fine motor co-ordination | Van Baar 1994 | Poor | 3 ½ years | Exposed children’s (n=22) median IBR fine motor co-ordination score was 3 (range: 1 – 5) vs. unexposed children (n=32) had a median score of 3 (range 1 – 5). |
|  | Wilson 1981 | Poor | 9 months | 75% of exposed children (untreated, n=29) showed poor IBR fine motor coordination vs. 82% of exposed children (Methadone, n=35) showed poor IBR fine motor coordination vs. 50% of unexposed children (n=55) showed poor IBR fine motor co-ordination. |
| Lack of Physical Development | Azuine 2019 | Fair | <6 years | Exposed children’s (n=3106) odds of lack of physical development was 1.8 (95% CI: 1.17, 2.79) compared to unexposed children (n=3106). |
|  |  |  | ≥6 years | Exposed children’s (n=2391) odds of lack of physical development was 0.62 (95% CI:0.26, 1.46) compared to unexposed children (n=2391). |
| Manual Dexterity Purdue Pegboard | Pulsifer 2008 | Good | 5 years | Exposed children’s (n=113) mean score was 3.8 (1.9) vs. unexposed children (n=31) had a mean score of 4.5 (2.6). |
| McCarthy Motor Scale | Ornoy 1996 | Fair | 3-6 years | Exposed children’s (n=20) mean score was 51.9 (8.2) vs. unexposed children (low SES, neglect) (n=16) had mean score of 46.5 (5.7) and unexposed children (n=23) had mean McCarthy Motor Scale score of 51 (7.5). |
|  | Hunt 2008 | Poor | 38.2 months | Exposed infant’s (n=67) score was 49.5 (8.7) vs. unexposed infants (n=44) had a score of 53.9 (8.3). |
|  | Moe 2002 | Fair | 4 ½ years | Exposed infant’s (n=64) McCarthy Motor Scale score was 48.9 (9.0) vs. unexposed infants (n=52) had a score of 55.8 (10.2). |
|  | Strauss 1979 | Poor | 5 years | Exposed infant’s (n=33) McCarthy Motor Scale score was 44.5 (9.3) vs. unexposed infants (n=30) had a score of 46.0 (10.7). |
| Motor function developmental disorder | Hall 2019 | Poor | >24 months | Out of children exposed to opioids without developing NAS after birth (n=473), 2.7% were diagnosed with motor function developmental disorder. 5.1% of exposed children with NAS after birth (n=138) were diagnosed with motor function developmental disorder. In unexposed children (n=14,933), 1.4% were diagnosed with motor function developmental disorder. |
| NEPSY hand positions | Konijnenberg 2013 | Poor | 4 ½ years | Exposed children’s (n=15) mean score was 8.80 (3.90) vs. unexposed children (n=15) had mean NEPSY hand positions score of 12.13 (4.53). |
| NEPSY Statue | Konijnenberg 2015 | Poor | 4 ½ years | Exposed children’s (n=35) mean score was 16.23 (7.86) vs. unexposed children (n=31) had mean NEPSY Statue score of 22.48 (7.08). |
| Object Motion Test | Melinder 2013 | Poor | 4 years | Exposed children’s (n=26) mean “looking time” domain score was 29.71 (2.1) vs. unexposed children (n=23) had a mean score of 30.25 (0.51).  Exposed children’s (n=26) mean “Saccades sec, slow” domain score on the Object Motion Test was 1.27 (0.6) vs. unexposed children (n=23) had a mean score of 1.22 (0.41).  Exposed children’s (n=26) mean “Saccades sec, fast” domain score on the Object Motion Test was 2.39 (0.9) vs. unexposed children (n=23) had a mean score of 2.04 (0.91).  Exposed children’s (n=26) mean “Smooth 6.78°/sec” domain score was 77.84 (12.2) vs. unexposed children (n=23) had a mean score of 79.49 (11.18).  Exposed children’s (n=26) mean “Smooth 13.2°/sec” domain score was 59.65 (15.07) vs. unexposed children (n=23) had a mean score of 69.77 (13.6). |
| Physical and Neurological Examination for Soft Signs (PANESS) | Grattan 1996 | Good | 8-11 years | Exposed children (n=35) showed a mean of 3.02 (2.85) errors across 5 gait tasks (Gaits and Stations Tasks) and a mean of 3.03 (1.74) gait tasks showing overflow vs. unexposed children (n=41) showed a mean of 3.37 (3.00) errors across 5 gait tasks and a mean of 2.80 (1.52) gait tasks showing overflow.  Exposed children (n=35) presented dysrhythmia in a mean of 1.41 (1.16) PANESS timed repetitive maneuvers and showed motor overflow in a mean of 1.21 (1.34) maneuvers vs. unexposed children (n=41) presented dysrhythmia in a mean of 1.03 (1.16) PANESS timed repetitive maneuvers and showed motor overflow in a mean of 1.36 (1.39) maneuvers.  Exposed children (n=35) presented dysrhythmia in a mean of 3.59 (1.34) PANESS timed alternating maneuvers and showed motor overflow in a mean of 1.94 (1.60) maneuvers vs. unexposed children (n=41) presented dysrhythmia in a mean of 3.46 (1.50) PANESS timed alternating maneuvers and showed motor overflow in a mean of 1.33 (1.26) maneuvers. |
| Quick Neurological Screening Test | Davis 1988 | Good | 6-15 years | Exposed children’s (n=28) Quick neurological screening test mean score for all items was 8.57(4.63) versus unexposed children’s mean score of 5.0. (SD=3.94) |

BSID: Bayley Scales of Infant Development, IBR: Infant Behavior Record, NEPSY: NEuroPSYchological Assessment, PDI: Psychomotor developmental index, PANESS: Physical and Neurological Examination for Soft Signs (PANESS)

^1^Same cohort as van Baar 1989. Thus, only the measures of van Baar 1994 is reported.

^2^Same cohort as Straus 1976. Thus, the 12 months measures of Straus 1979 with the larger sample size is reported.

^3^We calculated the average and standard deviation of sum of all Quick Neurological Screening Test items based on the findings reported in the study.

## Supplemental Figures

### Supplemental Figure 1. Proportion of studies assessing different types of prenatal exposure to opioids among all the included studies published from 1970-2020 based on subject recruitment period

**
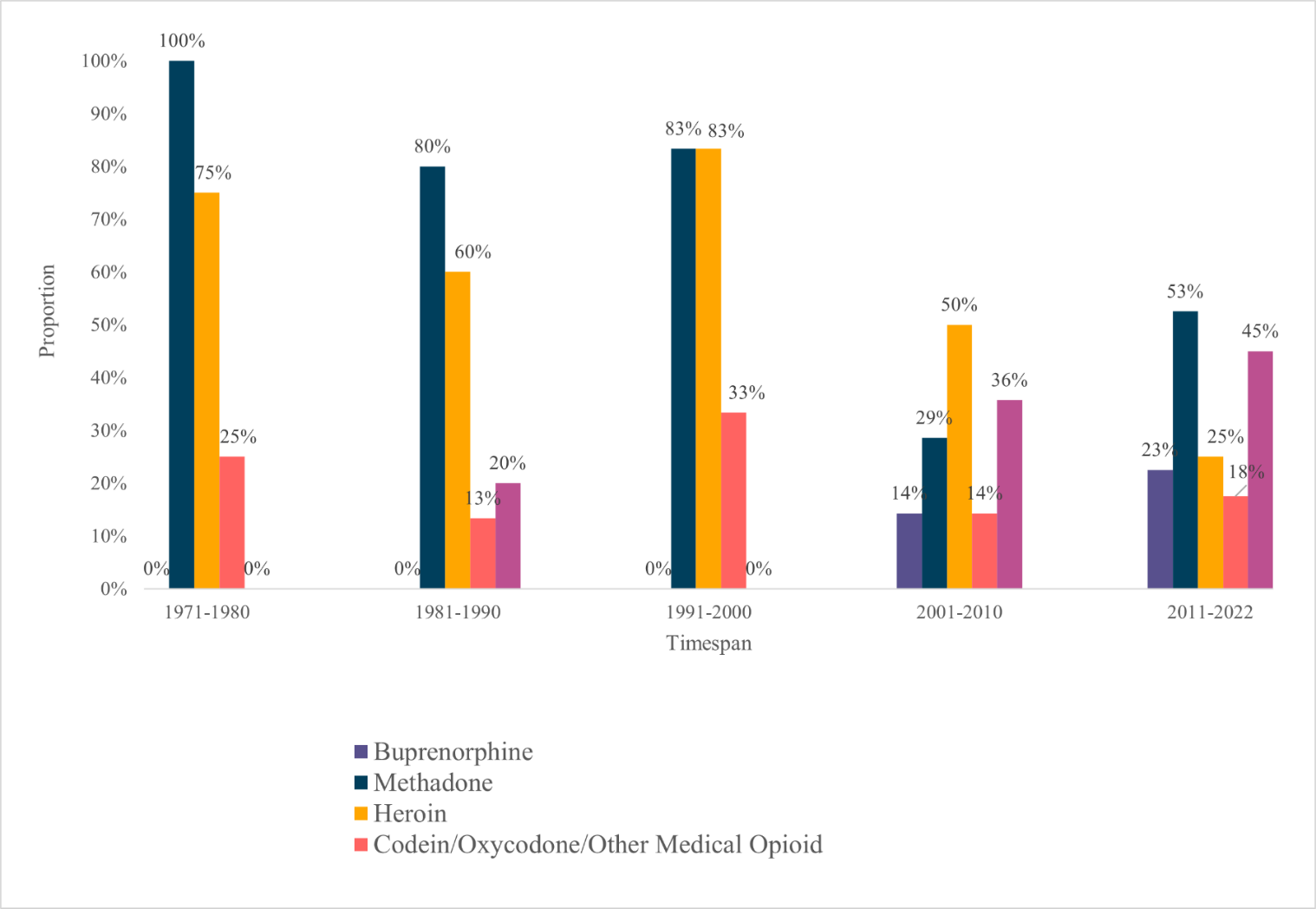
**

Note: More than one type of opioid might have been assessed in a single study
